# High-Entropy Catalysts for Biomass-Derived Chemicals Valorization: Mechanisms, Applications, and Opportunities

**DOI:** 10.1007/s40820-026-02192-x

**Published:** 2026-04-16

**Authors:** Fan Li, Longli Chen, Siwei Chen, Zhihui Ma, Qiang Wang, Fukuan Li, Feng Shen

**Affiliations:** 1 Agro-Environmental Protection Institute, Ministry of Agriculture and Rural Affairs, No. 31 Fukang Road, Nankai District, Tianjin, 300191 People’s Republic of China; 2 Key Laboratory of Rural Toilet and Sewage Treatment Technology, Ministry of Agriculture and Rural Affairs, No. 31 Fukang Road, Nankai District, Tianjin, 300191 People’s Republic of China

**Keywords:** High-entropy catalysts, Biomass valorization, Platform chemicals, Synergistic effects, Sustainable catalysis

## Abstract

The valorization of biomass-derived chemicals into high-value chemicals represents a crucial pathway toward a sustainable and low-carbon economy. However, the structural complexity and multifunctionality of these molecules demand catalysts capable of multisite activation and precise chemo-selectivity. High-entropy catalysts (HECs), which integrate five or more principal elements into a single phase, have recently emerged as a promising materials platform, offering tunable active sites, enhanced stability, and unique synergistic effects. This review provides a comprehensive and up‑to‑date overview of recent advances in HECs for the valorization of biomass‑derived chemicals. We first clarify the definition of high‑entropy materials and elucidate the relationships between the four core effects and catalytic performance. Subsequently, we systematically outline the key elements frequently incorporated in HECs, emphasizing their roles in modulating active sites and electronic structures. Design strategies, including component modulation, morphology/size regulation, defect engineering, and heterostructure construction, are discussed with a focus on synergistic mechanisms governing biomass conversion. The applications of HECs in major valorization reactions, including oxidation of 5-hydroxymethylfurfural (HMF), hydrogenation of HMF and furfural (FF), oxidative condensation of FF, depolymerization of lignin, and conversion of glucose and glycerol are detailed, highlighting clear correlations between catalyst structure and catalytic efficacy. Finally, we identify prevailing challenges and outline future research directions aimed at the rational design of next-generation HECs for efficient and selective biomass upgrading. This work aims to serve as a foundational reference and stimulate further innovation in the application of HECs in biorefinery and green chemistry.

## Highlights

- This review comprehensively summarizes the latest advances of high entropy catalysts (HECs) in biomass derived chemicals valorization, covering their core effects, elemental composition principles, structural design strategies, and typical application reactions.
- The unique advantages of HECs and their structure–activity relationships in biomass conversion are systematically clarified.
- Future directions of HECs for biomass-derived chemicals valorization are outlined, including precise design via computational-experimental integration, expansion of catalyst types and reaction scope, and exploration of novel structure regulation strategies.

## Graphical abstract

**Figure:**
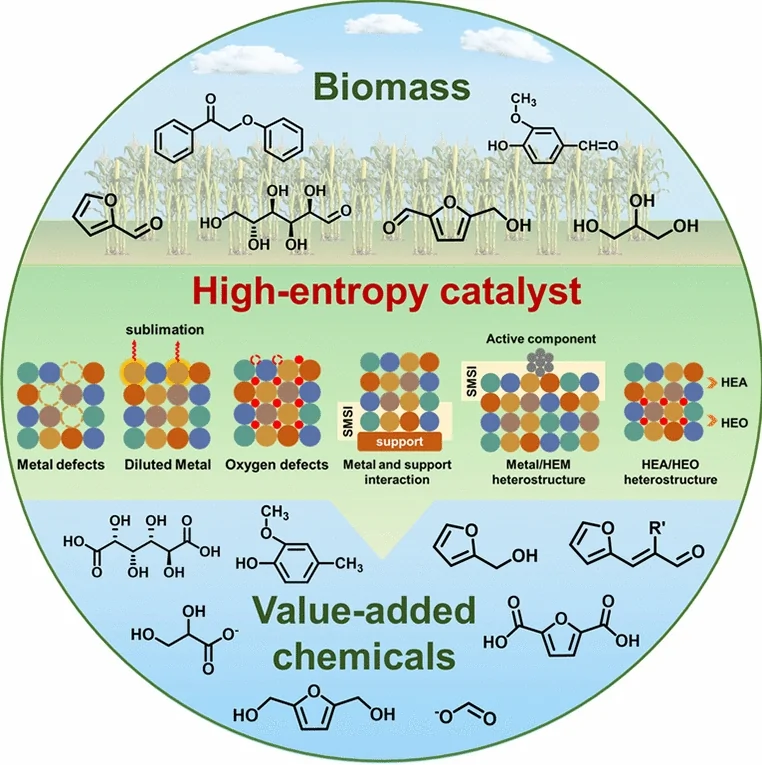

## Introduction

**Fig. 1:**
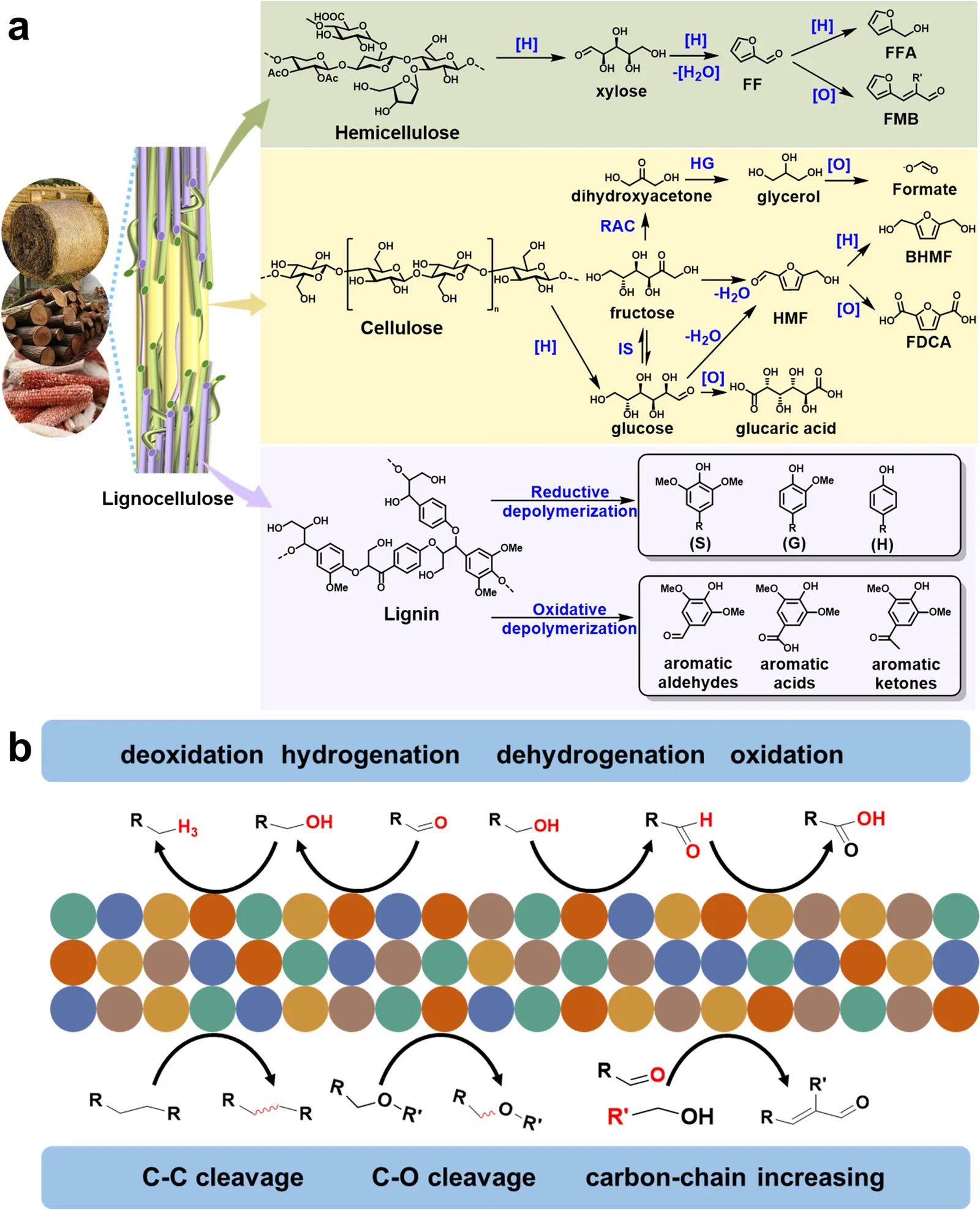
**a** Schematic of the lignocellulosic biomass structure and some representative conversion routes of cellulose, hemicellulose, and lignin into value added chemicals. IS: isomerization, RAC: retro-aldol condensation, HG: hydrogenation. **b** Biomass-derived chemicals valorization involves various catalytic reactions, including hydrogenation, dehydrogenation, C–C and C-O bonds cleavage, carbon chain increasing, etc. The multisites of HECs “cooperatively” catalyze the reactions

Against the backdrop of global climate and environmental crises driven by the overexploitation of fossil resources and soaring CO_2_ emissions, the shift toward a low-carbon circular economy has become a worldwide consensus, with the bio-based industry taking on a central role in this transition [1]. Biomass, the most abundant organic and renewable carbon resource in nature, can contribute to reducing dependence on fossil fuels. Lignocellulose is the most prospective biomass due to its carbon–neutral trait, cost efficiency, and ready attainability. It is primarily composed of three key biopolymers: cellulose (30%–50%), hemicellulose (20%–35%), and lignin (15%–30%) [2]. These components collectively constitute an intricate, cross-linked structural matrix in the cell walls of plant tissues. Cellulose is a polymer of glucose (GLU), hemicellulose is a heteropolymer containing primarily xylose, and lignin is a cross-linked, aromatic-based heteropolymer [3, 4]. With lignocellulose as the initial raw material, a broad array of routes for manufacturing value-added chemicals have been established, yielding more than 200 diverse types of high-value chemicals [5, 6]. Figure 1a presents the lignocellulosic biomass structure and some representative conversion routes.

The hydrolysis of cellulose into GLU serves as the initial step for cellulose valorization, a process that underpins the efficacy of all selective conversion pathways. GLU acts as a fundamental feedstock for the food manufacturing, pharmaceutical, and various other industrial domains. It can be also transformed into various value-added chemicals via both catalytic and electrocatalytic pathways [7]. GLU oxidation produces glyceric acid, glucaric acid, and formic acid while its hydrogenation produces sorbitol. The isomerization reaction enables the conversion of GLU into fructose, which serves as a critical platform molecule for biomass-based chemical production. Through retro-aldol condensation reaction, fructose can be converted to dihydroxyacetone and glyceraldehyde and then be further hydrogenated to glycerol (GLY) [3]. GLY oxidation produces chemicals such as glyceric acid, glycolic acid, and formic acid. The dehydration of GLU and fructose yields 5-hydroxymethylfurfural (HMF), a compound accepted as one of the most important and extensively studied platform molecules due to its capacity for conversion into a broad range of value-added chemicals such as 2,5-furandimethanol (BHMF) and 2,5-furandicarboxylic acid (FDCA) [8–10].

Acid-catalyzed hydrolysis of hemicellulose enables the extraction of diverse C5-sourced sugars (e.g., xylose and arabinose), which can subsequently be subjected to further upgrading for the synthesis of a broad range of value-added chemicals [11, 12]. Xylose hydrolysis and dehydration produce furfural (FF), which serves as the only precursor for all molecules that include a furyl, furfuryl, furoyl, or furfurylidene group in the chemical sector [13]. FF hydrogenation produces furfuryl alcohol (FFA) and its oxidative condensation increases carbon chain length. Lignin depolymerization yields aromatic monomers, which can be subjected to selective hydrodeoxygenation steps to afford a diverse range of value-added aromatic derivatives.

As stated above, biomass-derived chemicals have diverse functional groups, including aldehyde, ketone, carboxyl, hydroxyl, furan ring, C–C, and C-O linkages, etc. The valorization process involves various catalytic reaction types, including hydrolysis, dehydration, hydrogenation, oxidation, retro-aldol condensation, and carbon chain increasing, etc. (Fig. 1b). The intricate molecular architectures typically necessitate the synergistic interplay of multiple active sites to enable efficient activation and the attainment of high chemoselectivity. This has motivated the exploration of novel catalysts capable of delivering remarkable performance while meeting criteria for stability and products-orientated selectivity.

In this scenario, high-entropy catalysts (HECs) emerged as an intriguing family of catalytic materials that take advantage of multicomponent elemental modulation to access uncharted structure–property territories [14, 15]. Unlike traditional alloys generally dominated by one or two primary elements, HECs are constituted by five or more constituent elements. The multicomponent configuration contributes not only to phase stabilization but also endows tunable catalytic activity owing to abundant defects, diverse catalytic sites and electronic perturbations.

**Fig. 2:**
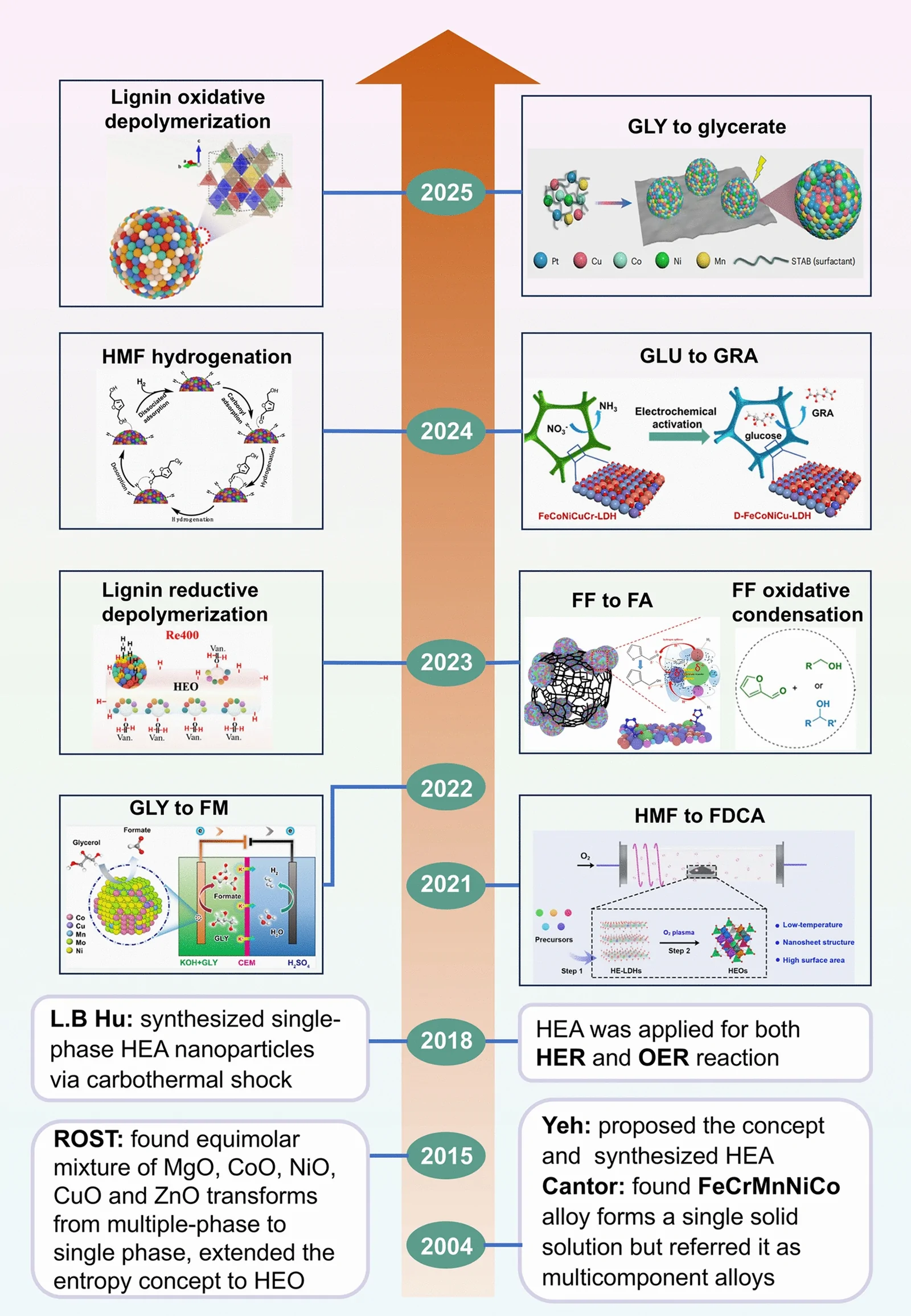
Historical timeline of HEMs and their application in biomass-derived chemicals valorization. Reproduced with permission [44]. Copyright 2021, John Wiley and Sons. Reproduced with permission [45]. Copyright 2022, American Chemical Society. Reproduced with permission [46]. Copyright 2023, Elsevier. Reproduced with permission [47]. Copyright 2023, American Chemical Society. Reproduced with permission [48]. Copyright 2023, John Wiley and Sons. Reproduced with permission [49]. Copyright 2024, Royal Society of Chemistry. Reproduced with permission [50]. Copyright 2024, Royal Society of Chemistry. Reproduced with permission [51]. Copyright 2025, Springer Nature. Reproduced with permission [52]. Copyright 2025, American Chemical Society

The development of HECs originated with the proposal of high-entropy alloy (HEA) concept in 2004. Subsequently, it has gradually expanded to various material systems, achieving continuous breakthroughs in size control, synthesis technology, and catalytic applications. We outlined a brief history of high-entropy materials (HEMs) and their application timeline for catalysis, with emphasis on biomass-derived chemicals conversion (Fig. 2).

### Concept Origin Stage (2004–2014)

The core of this stage was the birth of HEA concept, laying the theoretical foundation for the subsequent development of HECs. In 2004, Professor Yeh Jien-Wei’s research group proposed the concept HEA and synthesized series of HEAs. In the same year, Cantor’s team found FeCrMnNiCo alloy forms a single solid solution but referred it as multicomponent alloys [16, 17]. Their pioneer work breaks the traditional alloy design paradigm dominated by 1–2 principal elements. The HEA is defined as a new type of alloy containing 5 or more principal elements, with each element accounting for 5%–35% of the atomic fraction. Endowed with unique effects such as high mixing entropy and lattice distortion, these alloys exhibit excellent mechanical properties and corrosion resistance. However, research during this period mainly focused on exploring the basic characteristics of bulk HEAs, seldom delving into the catalytic field. Li et al. firstly synthesized PdNiCoCuFe and applied it as efficient electrocatalyst for methanol oxidation in 2014 [18].

### System Expansion and Practical Catalytic Breakthrough Stage (2015–2018)

In 2015, Rost et al. found equimolar mixture of MgO, CoO, NiO, CuO, and ZnO transforms from multiple-phase to single phase, the high-entropy concept extended from alloys to oxides [19]. This milestone marked the official birth of high-entropy oxides (HEOs), which possess potential for catalytic applications due to properties such as disordered cation distribution.

During this period, researchers established a library of high-entropy nanoparticles, providing a crucial foundation for the subsequent screening of high-entropy nanomaterials suitable for catalytic reactions. 2018 witnessed a pivotal breakthrough in the application of HEMs in catalysis. The successful synthesis of nanoscale HEAs officially established them as a major choice for high-performance catalytic materials. Hu’s team synthesized single-phase HEA nanoparticles for heterogeneous catalysis via carbothermal reduction [20]. This technological breakthrough was of great significance: it not only reduced the size of HEAs to the nanoscale but also integrated the high-entropy effect of HEAs with the surface effect of nanomaterials, significantly promoting the catalytic activity and compatibility of the materials. The nanoscale HECs are applicable to various catalytic reactions such as azo dyes degradation [21], ammonia oxidation [20], hydrogen evolution reaction (HER) [22], oxygen evolution reaction (OER) [23], and oxygen reduction reaction [24], truly initiating an upsurge in practical research on HEMs in the catalytic field.

### Diversified Application Stage (2019–Present)

In this stage, HECs have developed rapidly toward refined size control, diversified morphologies, and widespread applications. In terms of size, beyond the nanoscale, sub-nanoscale HEA particles and high-entropy single-atom catalysts (HE SACs) have been successfully developed, further optimizing catalytic active sites. In terms of morphology, researchers have designed HECs with various structures such as sub-nanowires, nanosheets, and aerogels to adapt to different catalytic reaction scenarios. Meanwhile, the material system of HECs has continued to expand, encompassing various compounds such as high-entropy carbides (HECBs) [25], high-entropy nitrides (HENs) [26], high-entropy sulfides (HESs) [27], high-entropy phosphides (HEPs) [28], high-entropy borides (HEBs) [29], high-entropy fluorides (HEFs) [30, 31], high-entropy layered hydroxides (HE-LH) [32], and high-entropy intermetallic (HEI) [33] catalysts. In terms of applications, HECs have demonstrated outstanding performance in numerous catalytic reactions, including alcohol oxidation [34], carbon dioxide reduction [35, 36], ammonia decomposition [37], nitrogen reduction reaction [38, 39], nitrate reduction reaction [40–42], and VOC removal from flue gas [43].

It was not until 2021 that Wang et al. reported a low-temperature plasma method for fabricating defect-rich HEO nanosheets and employed them for HMF electrooxidation [44]. It marks the first time that HECs were used for biomass upgrading. Since then, HECs were extensively utilized in a wide range of biomass conversion reactions, such as HMF and FF hydrogenation, FF oxidative condensation, GLY oxidation to formate and glycerate, GLU oxidation to glucaric acid, and lignin depolymerization.

In July 2023, Tong et al. published the first review about HEMs as catalysts for valorization of biomass and biomass-derived chemicals [53]. It focused on the general component, structure and catalytic conversion network of lignocellulosic biomass, inherent merits of HEMs and the potential biomass valorization reaction types without summarizing the element combinations and specific biomass valorization reaction due to limited publications (less than 10). Very recently, Srinivaas and co-authors reviewed the development of HEAs in biomass conversion, covered limited reactions and only focused on HEA [54].

This work aims to provide a comprehensive and the most-updated overview of recent progress on HECs for biomass-derived chemicals valorization. We begin with introducing the definition of HEMs and the four core effects, highlighting their relationship with catalytic reactions. We then summarize the highlighted elements frequently incorporated in HECs for biomass-derived chemicals valorization, emphasizing on each element’s characteristics in constituting HECs. Next, we discuss the design strategies of HECs, with a focus on how multimetallic synergy, defects and in situ reconstruction govern the biomass valorization reactions. Finally, we discuss the applications of HECs in various reactions, including HMF oxidation, HMF and FF hydrogenation, FF oxidative condensation, lignin depolymerization, GLU conversion and GLY oxidation. Taking these perspectives as a foundation, we outline the challenges and research opportunities in designing highly efficient HECs for biomass-derived chemicals valorization. The synthesis and characterization methods are the subjects of an enormous number of studies, which have been systematically overviewed by other excellent reviewers, so they are not elaborate further in this review [14, 55–62].

## Definition of HEM and the Four Core Effects

### Definition

When the concept of HEA was first put forward, Yeh and co-workers defined them as alloys consisting of five or more constituent elements in equimolar proportions. To broaden the horizons of alloy design, the definition was expanded: HEAs can also be formulated with principal elements whose individual concentrations fall within the range of 5 to 35 atomic percent [16]. As high-entropy concept expanded beyond HEA, HEM family covers a range of materials including metal oxides, carbides, nitrides and borides, etc. HEMs inherently exhibit pronounced configurational disorder, a direct consequence of the coexistence of multiple distinct elements occupying identical crystallographic lattice sites. Researchers have endeavored to delineate HEMs on the basis of their constituent element count and corresponding stoichiometric ratios. The configurational entropy change per mole (S_con_) is determined as follows [63, 64]:

$$\Delta {S}_{con}=-R\left[\left({\sum }_{i=1}^{n}{x}_{i}\mathit{ln}{x}_{i}\right)+\left({\sum }_{j=1}^{n}{x}_{j}\mathit{ln}{x}_{j}\right)\right]$$

where R is the gas constant and x_i_ and x_j_ are the molar fractions of cations and anions, respectively. n is the number of elements. For equimolar concentration, the formula can be abbreviated as S_con_ = RlnN. The configurational entropy change is 1.39R, 1.61R, and 1.79R for material contains 4, 5, and 6 elements, respectively. The configurational entropy change for HEM is more than 1.61R. Yeh and co-workers proposed that a value of 1.5R represents the critical threshold required to realize entropy stabilization.

It is important to state the definition of HEM again because some researchers think the constituent elements are required to achieve homogeneous dispersion across all dimensional scales, with atomic-scale uniformity being a critical prerequisite. The multicomponent alloys contain multiple phases should not be called HEAs. However, elemental spatial distribution nonuniformity and phases separation are ubiquitous in HECs for biomass valorization and other catalysis reactions [45, 51, 65]. If only alloys with single-phase solid solutions are HEAs, these HECs should be excluded from this review. Yeh and his colleagues clarified the core spirit of HEA is to highlight the elevated potential of entropy change in facilitating the formation of solid solution phases. The classification of a material as a HEA should be grounded in either its chemical composition or the configurational entropy calculated from composition [66].

### Four Core Effects and Their Relationships with Catalysis Performance

The four core effects including high-entropy effect, severe lattice distortion effect, sluggish diffusion effect, and cocktail effect were proposed in 2006 for describing and understanding the mechanisms of various special phenomena related to HEAs [66]. As outlined in the brief history of HEMs and their application timeline, the proposal of HEA concept and the four core effects are not designated for catalysis. The application of HEMs for catalysis lags far behind the proposition of the four core effects. Rethinking and clarifying the relationships of four core effects with catalysis performance is helpful for understanding HEMS as well as to aid the design of materials for catalysis.

1. The high-entropy effects:
  Entropy was initially a thermodynamic function and later evolved into a measure of the disorder degree in a system. In HEAs, when atoms of diverse species are blended in approximately equimolar ratios, the resultant atomic-scale disorder far exceeds that observed in conventional alloys. The pronounced mixing entropy herein promotes the formation of disordered solution states and weakens the propensity for atomic ordering and phase segregation. The actual number of phases present in HEAs is substantially lower than the maximum equilibrium phase count permitted by the Gibbs phase rule, and it also falls short of the upper phase limit achievable under the non-equilibrium solidification conditions [66]. The configurational entropy of HEMs contributes to its thermodynamic stability. By contrast, how well the material performs as a catalyst is determined by the kinetic processes involved in transforming reactants into final products. Professor Ozin pointed out whether a kinetic advantage comes from high-entropy effect or a combinatorial result of optimizing surface chemistry is an un-answered question [67]. Therefore, the claim that the enhancement of catalytic activity stems from the high-entropy effect is hasty.
2. The severe lattice distortion effect:
  HEM contains multiple principal elements, when atoms with varying sizes and different bonding energy are incorporated into an identical crystal framework, they induce local structural distortions. These distortions exert a substantial impact not only on mechanical characteristics such as strength but also on electrical conductivity, electron scattering and surface energy, which readily create or modify catalytic performance. The heterogeneous strain fields caused by the severe lattice distortion effect in HECs make them a promising candidate for catalysis [68]. Elastic strains enable the modulation of the adsorption free energy of alloy, thereby effectively lowering the energy barrier associated with catalytic reactions. What’s more, lattice strains can reduce the formation energy of structural defects, facilitating the generation of a large number of defects. Lattice strains also exert a regulatory effect on the position of the electron d-band center, which is a key factor that correlates with the adsorption energy of reactants and reaction intermediates during catalysis. For instance, the incorporation of La with large atomic radius into FeCoNiMnRu exacerbate lattice distortion, which caused shifts in the d-band centers and reduced the number of unpaired electrons, thereby retaining excellent activity and stability during catalytic water splitting [69].
3. The sluggish diffusion effect:
  The sluggish diffusion effect refers to the phenomenon that severe lattice distortion lowers atoms diffusion rate due to “traffic jam.” The sluggish diffusion effect benefits the decreased phase transformation rate and the maintenance of fine nanostructures, while also contributes to excellent stability. For example, the simulated diffusion coefficient of Ru in RuRhCoNiIr is two orders slower than that in Ru-Ni binary alloys [70]. In electrooxidation reaction, the mass activity of PtCuNiCoMn retained 90% of the initial value, while on PtCuNiCo it was 62% of the initial value [71].
4. The cocktail effect:
  The cocktail effect refers to the unexpected properties that emerge from elemental interactions, where the macroscopic properties not only come from each individual component and their inter-elemental interactions, but also additional indirect effects of the different components. For example, in FF hydrogenation on NiCoCuZnFe HEA, the electro-rich Cu repelled the furan ring for inhibiting over hydrogenation, Zn was the strongest adsorption sites of aldehyde group in FF, and Ni, Co, Fe dissociated hydrogen and transferred them to the aldehyde for selective hydrogenation [46]. The highly efficient FF conversion and almost 100% FFA selectivity originated from the cooperation of each individual element. However, in GLU electrochemical oxidation to glucaric acid on defect-rich D-FeCoNiCu-LDH, the inter-elemental interactions favor the reaction [49]. Cu-Co and Cu-Cu bridges promote dehydrogenation of the hydroxyl and hydrogen linked with carbon, respectively. Cu-Ni bridge facilitates the oxidation of aldehyde to carboxyl group. Moreover, the synergistic integration of various *d* orbitals with broadened distribution in HEAs intensifies *d-d* electron coupling effects, which yields a more extended electronic energy band width and consequently boosts the overall catalytic performance [72, 73].

**Fig. 3:**
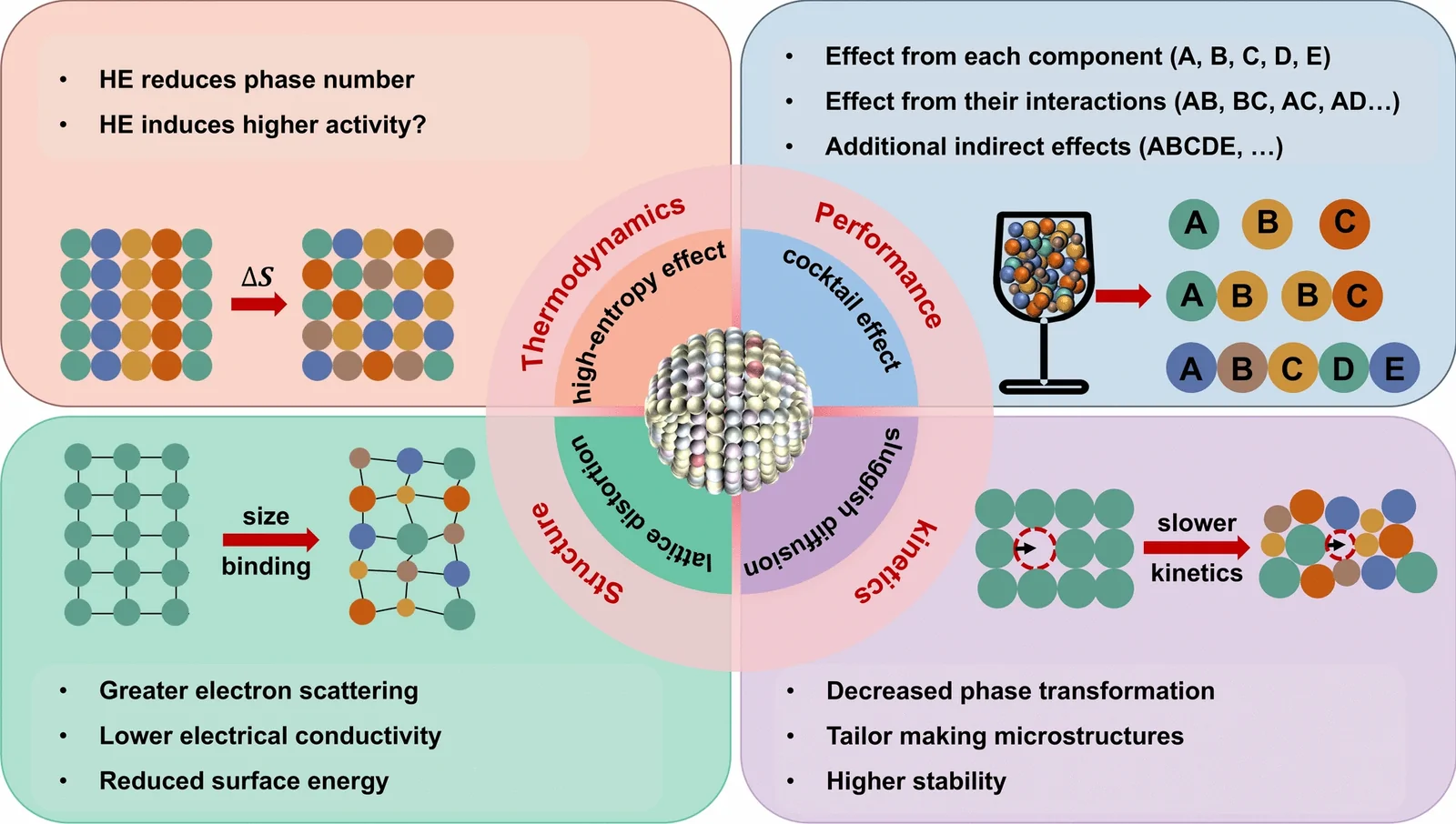
Core effects of HECs in catalysis reaction

The high-entropy effect, severe lattice distortion effect, sluggish diffusion effect and cocktail effect correspond to thermodynamics, structure, kinetics and performance, respectively (Fig. 3). Especially, the “unexpected” characteristics of cocktail effect definition make it cover all the factors and mechanism causing the deviations from a simple mixture.

## Highlighted Elements Frequently Incorporated in HECs for Biomass-Derived Chemicals Valorization

**Fig. 4:**
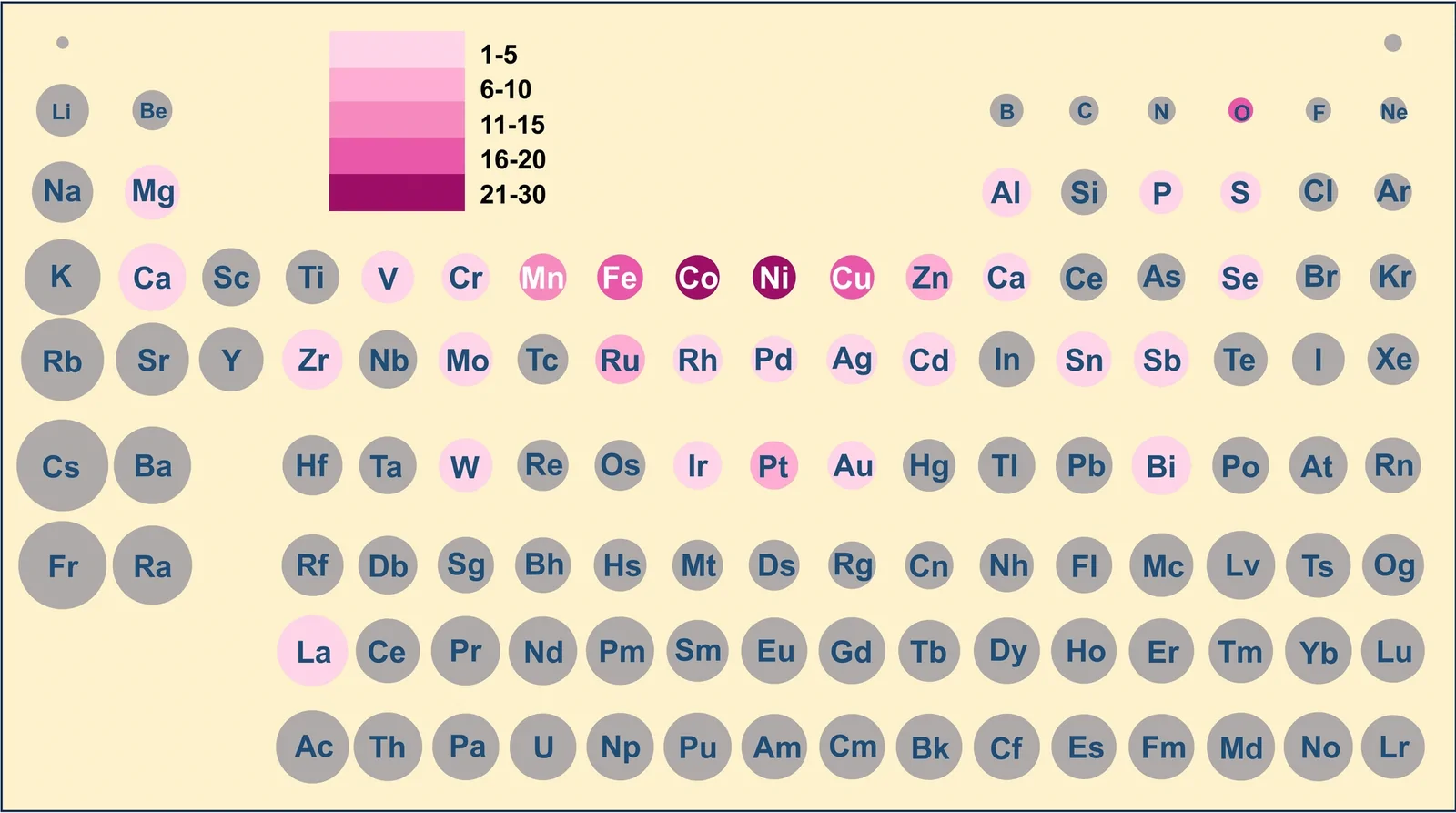
Highlighted elements incorporated in HECs for biomass-derived chemicals valorization. The size of a circle represents the relative size of atomic radii, as stated in Ref [95]. The gray circle indicates that the element has not yet been used to form a HECs for biomass-derived chemicals valorization. The purple depth indicates the use frequency of the element to form a HECs for biomass-derived chemicals valorization

The selection of constituent elements is critical to optimize HECs’ catalytic performance, as elements determine the type of active sites (redox, acid–base, or bifunctional), electronic structure, and stability under reaction conditions. We summarized the highlighted elements frequently incorporated in HECs for biomass-derived chemicals valorization in Fig. 4.

### Transition Metal Elements (Redox-Active Centers)

Transition metals are the core components of HECs, providing redox-active sites for hydrogenation, oxidation, hydrodeoxygenation, and other key reactions in biomass valorization. Their variable valence states enable efficient electron transfer between the catalyst and reactants, facilitating the activation of functional groups (e.g., C=O, C=C, C–C, –OH, and C–O–C).

The first-row transition metals were frequently used in HECs for biomass-derived chemicals valorization reaction, favored for their low cost, abundant reserves, and excellent hydrogenation/hydrodeoxygenation activity. Among them, Ni and Co were the most frequently used base transition metals in HECs. In electrocatalytic oxidation reaction, Ni and Co tend to form higher-valence species (oxyhydroxides, M-OOH) upon potential-driven oxidation, which serve as the active sites for biomass-derived chemicals oxidation [74, 75]. It was also supposed that Ni plays a crucial role in reducing reaction energy barrier for C–C cleavage [51, 76]. Integrating high-electronegativity Cu in HECs drives electron accumulation around Cu, which reduces adsorption energies of the intermediates, regulates reactant adsorption configuration, and inhibits by-product generation [46, 51, 77]. Cr and Zn have a high leaching potential during electrochemical reaction [49, 75]. The leaching of metals contributes to the formation of metal vacancies on the reconstructed HECs, favoring the exposed active sites and intermediate adsorption. For Zn-containing HECs, Zn can sublimate from the HECs core under high-temperature calcination, thus creating atomically dispersed Zn [46]. Zr, mainly in its + 4 oxidation state, exhibits strong oxygen affinity and tends to modulate the electronic distribution in HECs and induce the formation of OVs [48].

### Noble Metal Elements (Highly Active Sites for Selective Catalysis)

Noble metals are incorporated in HECs to boost catalytic activity and selectivity, especially for complex biomass conversion reactions requiring precise control of active site strength. Though costly, their loading in high-entropy systems leverages synergistic effects with base metals to reduce overall costs. Precious metals, including Au-, Ag-, and Pt-based metals (Ru, Rh, Pd, Ir, and Pt), were incorporated in HECs for biomass-derived chemicals valorization. Ru, Pt, and Pd are known for their superior hydrogenation activity and selectivity, so they are frequently used in HECs for hydrogenation reaction, such as HMF and FF hydrogenation [50, 78, 79]. Pd and Pt has a strong affinity toward furan ring in FF and HMF due to interaction between metal surface and π bond. Noble metals exhibit superb adsorption properties toward small organic molecules, which adjust HMF oxidation reaction products distribution. In all noble-metal HEA, the incorporation of Au or Ru in PtRhPdIr facilitates the HMF oxidation to DFF. If both Au and Ru are incorporated, the HMF oxidation to DFF and FFCA was inhibited [80]. Pt-based catalysts are the best candidate for GLY electrooxidation [51].

### Non-Metal Elements (Dopants for Electronic/Structural Tuning)

Non-metals (C, N, O, P, S, P, F) are often incorporated into HECs as dopants or part of the matrix, modifying the electronic structure of active metals, introducing defect sites and improving conductivity and stability. HECs such as HEO, HE-LH, HES, HESe, and HEP, formed by the combination of metals with inorganic elements (O, S, Se, P) were applied in biomass valorization. Unlike HEAs, which predominantly exhibit single-site occupancy within crystal structures, HEOs and other anion-containing HECs possess discrete cation and anion sublattices that engender enhanced structural diversity. Oxygen defects in HEO and HE-LH are the important adsorption site for most oxygenated reactants, which can efficiently promote the intrinsic activity [44]. The incorporation of Se, P, and S introduces more space for modulating the electronic properties and creating a rich coordination environment, which in turn enhances electrical conductivity, the formation of more defects, and facilitates surface reconstruction [81–83]. HESe, HEP, and HES readily reconstruct into oxyhydroxides, serving as the real active sites for electrochemical oxidation reactions, such as HMF and GLY oxidation [65, 75, 84].

The incorporation of C, N, and F in HECs which have demonstrated their superb activity and stability in catalysis reaction [85–87] is a further research orientation in biomass valorization reactions.

### Alkali Earth Metal Elements (Structure Regulators and Acid–Base Modifiers)

Alkali earth metals are usually not direct active sites but play critical roles in optimizing HEC structure and surface properties, enhancing catalytic activity and stability. In 2-phenoxy-1-phenyl-ethanone oxidation reaction, the control experiments revealed that alkaline earth metals (Ca and Mg) act a pivotal role in oxidative aerobic oxidative C–C bond cleavage reaction [88].

### Rare Earth Elements (Lattice Distortion Enhancers)

Rare earth elements are characterized by the large atomic radii and unique valence electron configurations [89]. They are incorporated in HECs to enhance lattice distortion, change electronic structure and improve catalytic stability. Studies found the incorporation of La in FeCoNiMnRu catalyst boosted the lattice distortion, caused shifts in the d-band centers and reduced the number of unpaired electrons [69]. Ce valued for its oxygen storage capacity and ability to form stable oxides, the reversible Ce^3+^ and Ce^4+^ pairs provide labile oxygen for forming oxygen vacancies and regulating metal–oxygen bond strength [90]. The pronounced oxygen affinity of Y facilitates the formation of stable Y–O moieties. These species exert a repulsive interaction with oxygen-containing intermediates, thereby efficiently inhibiting the excessive deposition of surface oxides and enhancing the antioxidation performance [91, 92]. La was alloyed with Mg, Fe, Mn, and Cu for FF oxidative condensation, represented the only rare earth element containing HEA combinations for biomass-derived chemicals valorization [47].

Other main group element such as Al, Ga, Sn, Sb, and Bi were also incorporated in HECs for biomass-derived chemicals valorization. Gallium (Ga) features a low mixing enthalpy when alloyed with the majority of metallic elements, and the alloy system presents minimal mixing enthalpy as well as a low Gibbs free energy throughout the alloy formation process [93]. When Ga was alloyed with Fe, Co, Ni, Cu, Pt, the HEA nanoparticles presented a diameter of 1.25 nm without agglomeration [94].

## Design Strategies of HECs

The multielement collaboration is the most significant effect in HECs. By screening different elements and tailoring compositional ratios, the multisites of HECs “cooperatively” catalyze the biomass-derived molecules. For example, in FF hydrogenation on NiCoCuZnFe HEA, Zn acted as the adsorption sites for aldehyde group, electron-rich Cu repelled the furan ring from over hydrogenation, other metals dissociated hydrogen and transferred them to the aldehyde for selective hydrogenation [46]. Even for the same atom, different surroundings will make the atom have different local density of states [96]. The atom loses its elemental identity but broaden the valence band spectrum, which is beneficial for catalysis reaction. In some cases, the high-entropy environment makes the active atoms maintain at high activity state [51].

Apart from component modulation, morphology and size regulation are also prevalent strategies. HECs with distinctive morphologies, including nanoparticles, nanosheets, nanofibers, hollow arrays, and mesoporous nanoplates were fabricated for biomass valorization. Particle size minimization leads to a dramatic rise in the ratio of exposed surface atoms, as their population grows exponentially with decreasing particle dimensions.

**Fig. 5:**
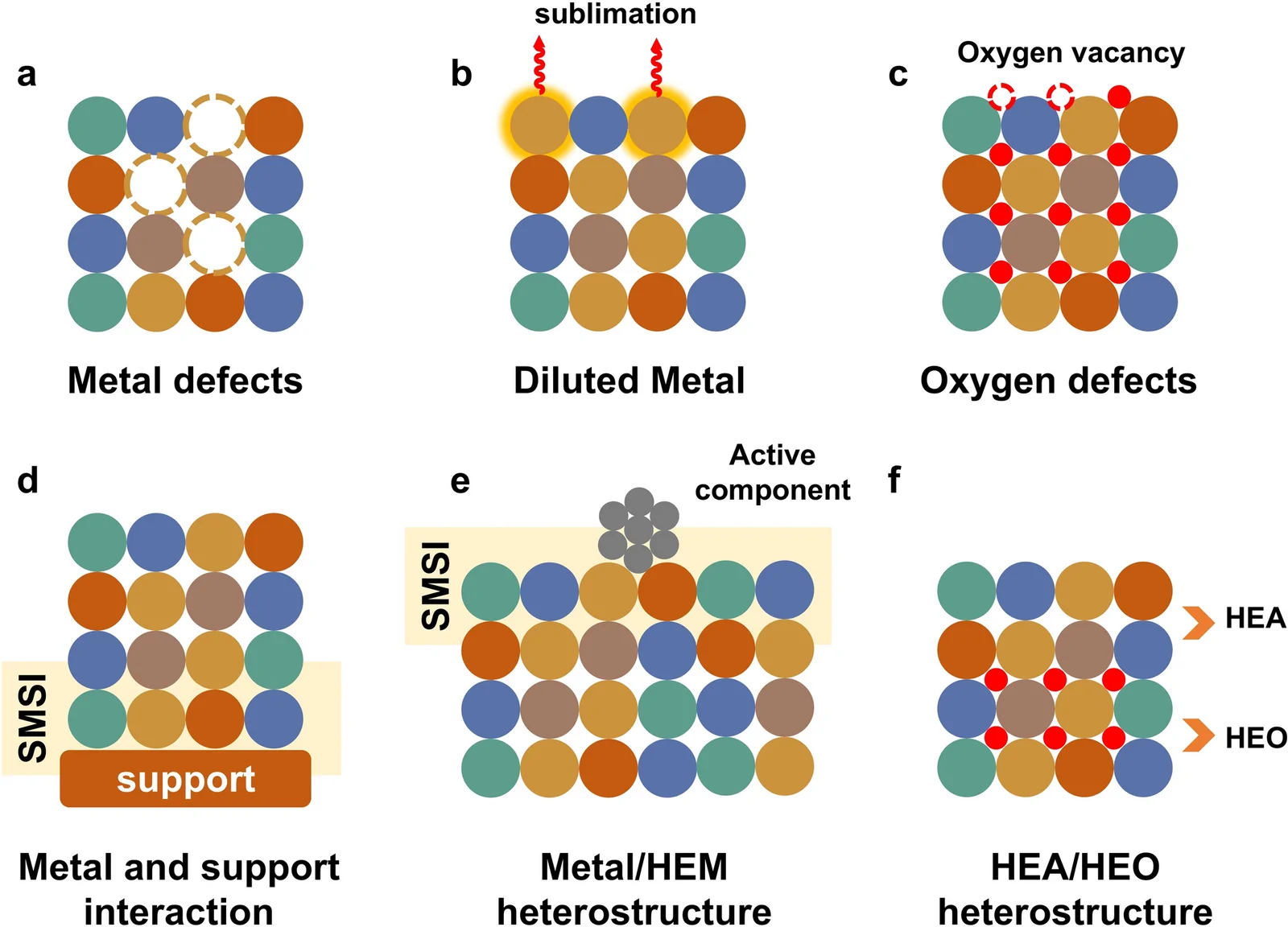
Applied design strategies of HECs for biomass valorization: **a** Metal defects, **b** diluted metal, **c** oxygen defects, **d** metal and support interaction, **e** metal/HEM heterostructure, **f** HEA/HEO heterostructure

The lattice distortion effect in HECs induces structure defects such as twinning, dislocations, and stacking faults. Rational introduction and regulation of such defects serve as a potent strategy to modulate the electronic configuration, promote the exposure of active sites, and facilitate reaction kinetics, thus optimizing the catalytic activity of HECs. As shown in Fig. 5a–c, metal defects, diluted metal, and oxygen defects were fabricated by metal dissolution, thermal reduction, and strain engineering [44, 46, 49].

Metal defects in catalyst induce electronic delocalization and create unsaturated coordination sites, which enhance the adsorption of reactant and activation of reactive species on active sites [97]. The coordination configurations of Co, Ni, and Cu underwent significant modification when Cr was completely removed from FeCoNiCuCr-LDH by electrochemical activation. Compared with FeCoNiCuCr-LDH, the Cr-defect FeCoNiCu-LDH exhibited a higher density of defects and a more disordered structural feature, which is conducive to exposing additional active sites for GLU oxidation reaction [49].

Dilute alloys have emerged as extensively studied catalytic materials, featuring atomically dispersed active sites that enable selective adsorption of reactants. These unique atomic configurations not only effectively modulate the surface atomic arrangement of HEAs but also fine-tune their d-band centers, thereby maximizing both catalytic activity and product selectivity. Under high-temperature treatment, Zn sublimated from the NiCoCuZnFe HEA core [46]. The content of zinc was only 0.45% in the NiCoCuZnFe/C-800 catalyst. The diluted Zn dispersed atomically within the HEA matrix and presented the strongest aldehyde group adsorption performance. The ultra-dilute HEA catalyst exhibited highly active hydrogenation of FF and nearly 100% selectivity of FFA.

Oxygen defects in HEOs or HE-LH serve as dual-functional sites: They act as Lewis acid sites to anchor oxygenated groups (e.g., aldehyde/hydroxyl groups) and facilitate electron transfer between the catalyst and reactants [98, 99]. In electrochemical oxidation reaction, the HEOs nanosheets prepared by low-temperature plasma strategy with rich oxygen vacancies delivered higher intrinsic activity for HMF oxidation than that of HEOs prepared by high-temperature method [44]. In FF hydrogenation and vanillin hydrodeoxygenation reaction, the efficient adsorption of the aldehyde group in reactant was proved primarily occurred on the oxygen vacancies [48, 100].

Disparities in atoms compatibilities and lattice parameters between heterophases induce the redistribution of electrons at heterogeneous interfaces, thereby facilitating electron transfer and boosting the intrinsic catalytic activity. In FeCoNiCrMnS_2_ HES nanoparticles, Ni/Cr and Co/Fe/Mn sperate phases were formed on the surface due to the different metal atoms compatibilities [65]. Loading HECs nanoparticles onto support could induce strong metal and support interactions (SMSI) (Fig. 5d). At the HECs/carbon heterointerface, the empty 3*d* orbitals of transition metal species in HEMs nanoparticles are capable of hybridizing with the 2*p* orbitals of carbon, which triggers the transfer of electrons from HEMs to the carbon support and tunes the electronic structures of the two components [101]. The SMSI adjusted the electron-environment of TiO_2_ and HEA interface in PdPtRuMoZn-TiO_2_, causing OVs-rich TiO_2_ and reduction of the horizontal configuration for FF, resulting superb FF conversion and FFA selectivity [100]. HEMs themselves can also act as support (Fig. 5e). Pt-HEO and CuS@HE-LH with heterostructure were fabricated for HMF oxidation [102, 103]. The SMSI induces electronic modulation and thereby promotes the formation of active oxyhydroxides species. The HEA/HEO catalyst were fabricated by thermal reduction of HEO (Fig. 5f) [48]. The synergy between OVs-rich HEO matrix and HEA particles with superb hydrogenolysis ability enables the heterogeneous catalyst to exhibit excellent hydrodeoxygenation activity.

## Applications

We summarized the HECs types, element combinations, synthesis methods, morphologies, and the biomass-derived chemicals valorization reaction types in Table 1. We categorized the applications to different reactions, with emphasis on the relationship of HECs structures with their catalysis performance.

**Table 1:** Overview of HECs for biomass-derived chemicals valorization

Catalyst type	Material	Catalytic reaction	Type	Synthesis method	morphology	Performance	References
HEO	(FeCrCoNiCu)_3_O_4_	HMF → FDCA	Dehydrogenation	Low-temperature plasma	Nanosheets	Over 90% yield and FE	[44]
HEA	PtRuAgCoNi	HMF → FDCA	Dehydrogenation	Solvothermal method	Nanoparticles	1.4 V, 55.2% conversion, 46.0% FDCA yield, 45.1% FE	[104]
HEA	PtRhPdIrRuAu	HMF → FDCA, HMFCA, DFF, FFCA	Dehydrogenation	Co-reduction	Nanoparticles	1.66 V	[80]
HE-LH	CoNiMnCuZnCdMg-LH	HMF → FDCA	Dehydrogenation	MOF-templated strategy	Hollow array	1.38 V, 100% conversion, 99% selectivity	[74]
HEO	(CoNiMnCuZn)_3_O_4_	HMF → FDCA	Dehydrogenation	Basic carbonate salts-oxide calcination	Mesoporous nanoplates	1.435 V, 99.3% conversion, 97.7% selectivity	[105]
HEA	AuCuAgPdNi	HMF → FDCA	Dehydrogenation	Wet chemical method	Nanowires	1.60 V, 95.5% yield, 98.4% selectivity	[72]
HEA	AuCuAgRuNi	HMF → FDCA	Dehydrogenation	Wet chemical synthesis	Nanofibers	1.50 V, 97.15% yield, 98.40% selectivity	[73]
HEA	CoNiMnMoPd	HMF → FDCA	Dehydrogenation	Nanofiber-mediated confined growth	Nanoparticles	1.53 V, 92.5% FE, 89.5% conversion, 95.8% selectivity	[106]
HE-LH	NiCoFeMnAl-LH	HMF → FDCA	Dehydrogenation	Hydrothermal	Urchin-like morphology	1.43 V, 100% conversion, 99.09% selectivity, 96.9% FE	[107]
HES	MoCrCoNiZn-S	HMF → FDCA	Dehydrogenation	MOF-derived strategy	Amorphous Particles	98% conversion, 98% yield, 94% FE	[75]
Pt-HEO	Pt- -Zn(AlCrMnFeCo)_2_O_4_	HMF → FDCA	Dehydrogenation	Calcination and reduction	Particles	1.38 V, 96.8% selectivity, 99.8% FE	[102]
CuS@HE-LH	CuS@NiFeZrWCe-LH	HMF → FDCA	Dehydrogenation	Template strategy	Crystalline-amorphous heterostructure	1.70 V, 98.04% conversion, 95.60% yield, 97.34% FE	[103]
HEA	Fe_1_Co_1.5_Ni_1_Cu_1.5_Pt_1.5_	HMF → FDCA	Dehydrogenation	Annealing and thermal reduction	Nanoparticles	0.5 MPa O_2_, 120 ℃, 6 h, 100% conversion, 94.8% yield	[108]
HEA	Fe_1_Co_1_Ni_1_Cu_1_Pt_2_Ga_1_	HMF → FDCA	Dehydrogenation	Impregnation-calcination	Nanoparticles	0.5 MPa O_2_, 100 ℃, 4 h, 100% conversion, 98.6% selectivity	[94]
HEA	FeCoNiCuRu	HMF → BHMF	Hydrogenation	Tannic acid assisted thermal reduction	Nanoparticles	5 MPa H_2_, 90 ℃, 2 h, 98.5% conversion, 99% selectivity	[50]
HEA	NiCoCuZnFe	FF → FFA	Hydrogenation	High-temperature calcination	Nanoparticles	3 MPa H_2_, 90 ℃, 9 h, 87.3% conversion, 100% selectivity	[46]
HEA	PtCoNiCuZn	FF → FFA	Hydrogenation	MOFs-template pyrolysis	Nanoparticles	IPA as the hydrogen donor, 100 ℃, 15 h, 94% conversion, 100% selectivity	[79]
HEA	PdPtRuMoZn	FF → FFA	Hydrogenation	High-temperature calcination	Nanoparticles	2 MPa H_2_, 30 ℃, 90.82% conversion, 91.3% selectivity	[100]
HEA	FeCoNiCuPd	FF → FFA	Hydrogenation	Electrochemical deposition	Particles	25 mA/cm^2^, 4 h, 100% conversion, 89% selectivity	[78]
HEA	MgLaFeMnCu	FF → FMB	Increasing of carbon chain	Oil phase wet chemical	Particles	0.3 MPa O_2_, 80 ℃, 2 h, 99.7% conversion, 93.8% selectivity	[47]
HEO	MnFeCoNiCuO_x_	FF → FMB	Increasing of carbon chain	self-propagation processes	Particles	0.3 MPa O_2_, 80 ℃, 2 h, 99.0% conversion, 91.5% selectivity	[109]
HEO/HEA	NiCuZnFeAl/NiZnCuFeAlZrO_x_	VAN → MMP	Hydrodeoxygenation	Metals hydroxides calcination and reduction	Particles	2 MPa H_2_, 120 ℃, 2 h, 100% conversion, 95% selectivity	[48]
R-HEO	MgCuMnFeCo-R	2-phenoxy-1-phenyl-ethanone → phenol and benzoic acid	Oxidative C–C bond cleavage	In situ high-temperature treatment	Particles	0.3 MPa O_2_, 140 ℃, 4 h, 94% conversion, 81.5% phenol selectivity and 99.0% benzoic acid selectivity	[88]
HEO	(AlCrFeCoCu)_3_O_4_	lignin → aromatic monomers hemicellulose → aliphatic acids	Oxidation Catalytic Fractionation	Solvothermal method	Nanoparticles	0.3 MPa O_2_, 160 ℃, 30 min, lignin into aromatic monomers with a yield of 42 wt %, total carboxylic acid yield of 150 wt % relative to the original hemicellulose content	[52]
HE-LH	D-FeCoNiCu-LH	GLU → GRA	Hydroxyl and aldehyde groups oxidation	Electrochemical deposition	Nanosheets	1.22 V, 100% conversion, 90% selectivity	[49]
HEA	CoNiCuMnMo	GLY → FM	C–C cleavage hydroxyl oxidation	pyrolysis reduction	Nanoparticles	1.27 to 1.47 V, over 90% FE of FM	[45]
HESe	(CoNiCuMnMo)Se	GLY → FM	C–C cleavage hydroxyl oxidation	Hydrothermal process	Nanosheets	1.57 V, selectivity of 88%	[84]
HES	FeCoNiCrMnS_2_	GLY → FM	C–C cleavage hydroxyl oxidation	Hydrothermal method	Nanoparticles	1.20 to 1.45 V, over 90% FE of FM	[65]
HEP	FeCoNiCuP	GLY → FM	C–C cleavage hydroxyl oxidation	Electrodeposition method	nanosheet arrays	1.4 V, 87.9% selectivity	[110]
HE-LH	FeCrCoNiCu-LH	GLY → FM	C–C cleavage hydroxyl oxidation	Hydrothermal	nanosheets	1.42 V, 92.9% FE	[111]
HEA	FeCoNiCrM (M: Pt, Pd, Ir)	GLY → FM	C–C cleavage hydroxyl oxidation	Electrospinning with calcination	Nanoparticles	1.57 V, 86.2% FE	[112]
HEA	NiCuCoMnCr	GLY → FM	C–C cleavage hydroxyl oxidation	Electrospinning with calcination	Nanofiber	1.45 V, 97.6% FE	[113]
HEA	PtCu@PtCuCoNiMn	GLY → glycerate	Hydroxyl oxidation	Wet chemical coupled with electrochemical activated	Nanoparticles	0.8 V, 75.2% selectivity	[51]

### HMF Oxidation

Selective oxidation of HMF to FDCA is attracting growing attention, given that FDCA ranks among the twelve key priority chemicals in green chemical industries [114]. Additionally, it stands as the most thoroughly researched reaction in the context of biomass valorization using HECs.

**Fig. 6:**
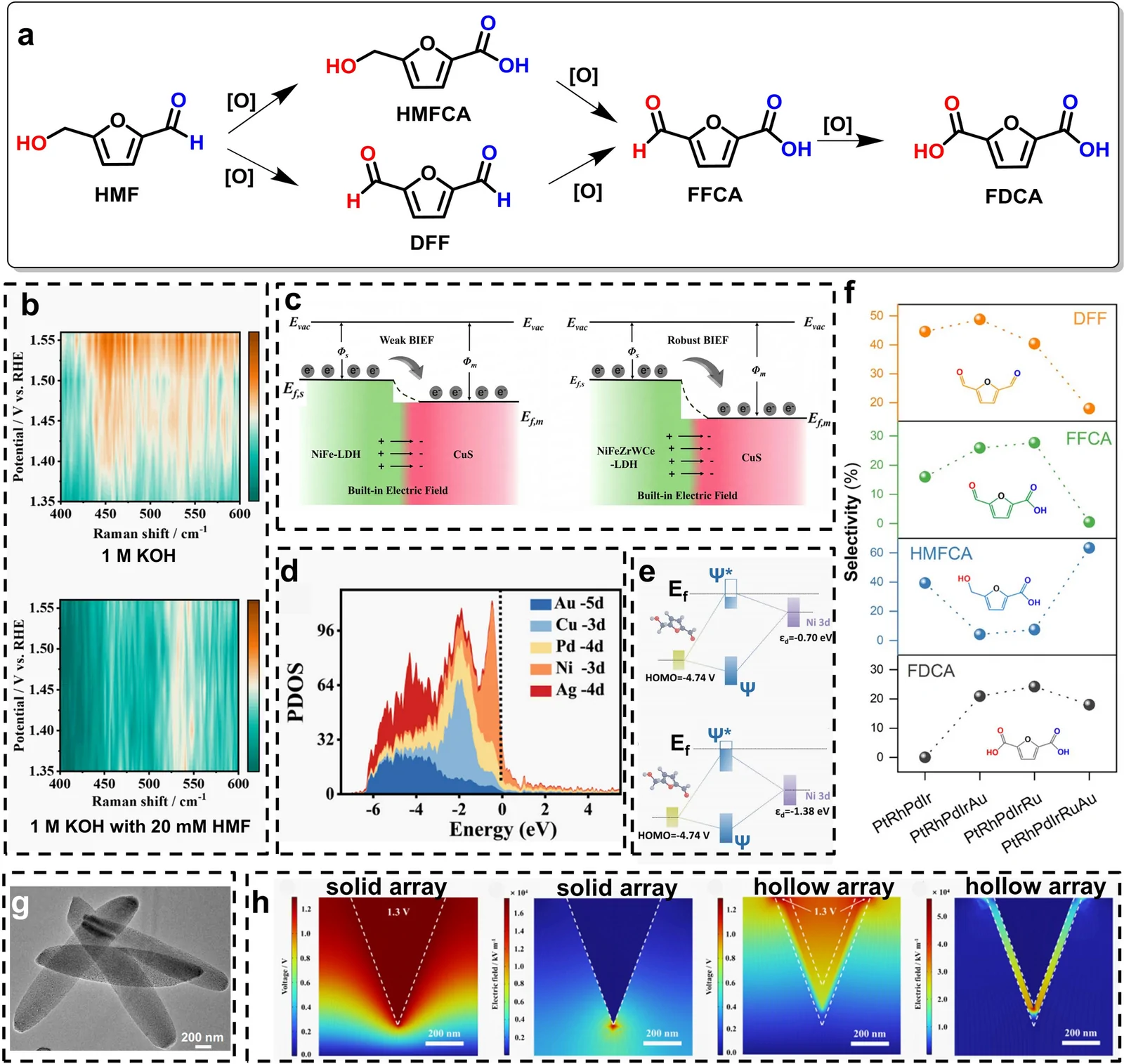
**a** Reaction pathway of HMF oxidation to FDCA. **b** In situ Raman spectra of CC@CoNiMnCuZnCdMg-LH arrays in 1 M KOH without and with 20 mM HMF. Reproduced with permission [74]. Copyright 2024, American Chemical Society. **c** Energy band diagram of CuS@NiFe-LDH and CuS@NiFeZrWCe-LDH. Reproduced with permission [103]. Copyright 2025, John Wiley and Sons. **d** PDOS of AuCuAgPdNi NWs. Reproduced with permission [72]. Copyright 2025, Elsevier. **e** Coupling between the HOMO of HMF and the Ni 3*d* band of HEA (up) and Ni (down). Reproduced with permission [106]. Copyright 2025, John Wiley and Sons. **f** Product selectivity and relative reaction rates of each step in HMF electrocatalytic oxidation reaction on different alloys. Reproduced with permission [80]. Copyright 2023, Royal Society of Chemistry. **g** TEM images of mesoporous (CoNiMnCuZn)_3_O_4_ nanoplates. Reproduced with permission [105]. Copyright 2024, Springer Nature. **h** Simulated electric potential distribution and corresponding electric field distribution images of the solid and hollow leaf-like arrays. Reproduced with permission [74]. Copyright 2024, American Chemical Society

HMF contains aldehyde (-CHO) and hydroxyl groups (OH) in its molecular structure. The oxidation of these two functional groups results in the forming of a range of valuable chemical products or intermediates which makes the HMF oxidation reaction (HMFOR) proceed in two possible pathways (Fig. 6a) [115, 116]. In pathway (1), aldehyde is initially oxidized to carboxylate (COOH) with the formation of 5-hydroxymethyl-2-furancarboxylic acid (HMFCA). Then, the consecutive oxidation of hydroxymethyl yields formyl-2-furancarboxylic acid (FFCA) and FDCA. In pathway (2), the hydroxymethyl is initially oxidized to aldehyde with the formation of 2,5-diformylfuran (DFF). Then, the two aldehyde groups are oxidized consecutively to form FFCA and FDCA.

The electrooxidation of HMF typically involves a coupled electrochemical-chemical oxidation mechanism [117, 118]. Typically, the catalysts are initially reconstructed with the transition of low valence metal species to higher-valence species (oxyhydroxides, M-OOH) upon potential-driven oxidation. Subsequently, the oxyhydroxides act as the active sites for adsorbed HMF oxidation while they were concomitantly reduced to lower species. The lower valence metal species are then reoxidized to oxyhydroxides, thus completing the redox cycle. In situ Raman spectroscopy is often carried out to explore the surface dynamic evolution of HECs in electrochemical oxidation reaction. As presented in Fig. 6b, a pair of Raman peaks at about 450 and 550 cm^−1^ assigned to vibration modes of NiOOH species is intensified when potential moves positive for CC@CoNiMnCuZnCdMg-LH arrays in 1.0 M KOH. On contrary, these characteristic peaks are not obvious in the presence of 20 mM HMF, demonstrating the in situ generated oxyhydroxides act as the active species for HMFOR [74].

A high-entropy amorphous MoCrCoNiZn-S HES was fabricated for HMFOR [75]. The in situ Raman confirmed amorphous structure favors the in situ generation of abundant oxyhydroxides driven by high-potential in alkaline electrolytes, while in HMF containing system the oxyhydroxides were consumed for dehydrogenation. Strategic engineering of the surface electronic structure in HECs is favorable for enhancing HMF adsorption and promoting surface reconstruction to form active M-OOH species. In Pt-decorated HEO catalyst, the SMSI modulates the electronic structure of the HEO catalyst which promotes the adsorption of OH^−^ and their transition into active oxyhydroxides species [102]. The oxyhydroxides species act as the nucleophiles that attack the aldehyde group. The Pt-decorated HEO catalyst achieved a 99.8% FE and 96.8% selectivity for FDCA. Yang et al. synthesized a novel CuS@NiFeZrWCe-LDH catalyst [103]. The work function difference between CuS and high-entropy NiFeZrWCe-LDH is much larger than that between CuS and NiFe-LDH (Fig. 6c). The stronger built-in electric field (BIEF) at the heterojunction interfaces in CuS@NiFeZrWCe-LDH favors efficient charge transfer from NiFeZrWCe-LDH to CuS, which induces surface reconstruction to generate high-valence M-OOH species. Furthermore, the multimetallics sites in HE-LH fine-tune the hydroxyl oxidation through the d-d electron interactions.

The electrochemical-chemical oxidation mechanism involves continuous electro transfer and phase transitions, which can provoke negative impact on reaction rates and stability [119, 120]. Zhu’s team found high-entropy environment in CoNiMnMoPd HEA enabled Ni maintain a high-valence state, acting as the adsorption sites for HMF. The dehydrogenation primarily occurs at adjacent site to Ni. It implies HMFOR in Ni-based HEA proceeds in electrochemical oxidation mechanism. This catalyst exhibited a HMF conversion of 89.5% and 92.5% FE for FDCA. Furthermore, it demonstrated persistent stability over 100 h operation [106].

The position of the d-band center determines the interaction strength between the catalyst surface and reactant molecules, thereby affecting the activity and selectivity of catalysis [121]. When the d-band center shifts toward the Fermi level, the interaction is enhanced, which facilitates the progression of the reaction. The combination of broader distribution of various *d* orbitals in HEA promotes the d-d electron interactions, resulting in wilder electronic width and enhanced catalytic performance. The metals with higher *d*-band center present stronger HMF adsorption [74]. Yin’s group synthesized AuCuAgPdNi and AuCuAgRuNi for HMF electrocatalytic oxidation [72, 73]. The high-resolution TEM images revealed the as-prepared HEAs possessed plenty structural defects, including grain boundary, twin boundary, amorphous sites, and atomic step. Specially, the significant overlap of *d* orbitals among the five elements implies an enhanced electron transfer efficiency in HEAs, thus achieving complete HMF oxidation and 99% FDCA selectivity (Fig. 6d). In CoNiMnMoPd HEA, the high-entropy environment makes the d-band center of Ni shift upward (Fig. 6e), facilitating the adsorption of HMF at Ni sites [106]. For HEO catalyst, the same phenomenon was also detected. The d-band center of Co in (CoNiMnCuZn)_3_O_4_ is closer to the Fermi level than that in Co_3_O_4_, indicating a stronger capacity of adsorbing intermediates on HEO. The peak patterns of Co PDOS are more numerous and broader in the HEO catalyst than in Co_3_O_4_ [105].

For high-entropy layered hydroxides (HG-LH), the lattice oxygen acts as a pivotal role in the electrocatalytic reactions. Liao et al. found that the surface high-entropy environment in NiCoFeMnAl HE-LH regulates the *p*-band center of lattice oxygen downward [107]. The higher gap between the p-band center and the Fermi level implies a weaker adsorption toward HFM. The excessive adsorption strength diminishes the active sites and induces surface carbon deposition, which caused lower HMFOR activity and FDCA selectivity in NiCoFe-LH.

Regulating mole ratios of different metals in non-equimolar HEA catalysts has a strong influence on product selectivity. Fe_1_Co_1_Ni_1_Cu_1_Pt_1_ and Fe_1_Co_1.5_Ni_1_Cu_0.75_Pt_1_ presented FDCA yield of 40% and 65%, respectively. Under the same reaction conditions, absolute HMF conversion and 94.8% FDCA yield was achieved on Fe_1_Co_1.5_Ni_1_Cu_1.5_Pt_1.5_ [108]. When Ga element was incorporated into Fe_1_Co_1_Ni_1_Cu_1_Pt_1_, the reaction rate constant of the rate-determining step was boosted by two orders of magnitude [94].

In most studies, HECs constitute all non-noble metals or non-noble metals alloyed with minor noble metals for reducing the usage of precious metals. Peng et al. synthesized all noble-metal based HEAs for HMFOR (Fig. 6f) [80]. The senary HEA catalyst exhibited the highest current density among the prepared alloys and the product distribution varies a lot depending on the composition. The PtRhPdIr quaternary alloy favors the initial oxidation of aldehyde and hydroxyl groups, forming HMFCA and DFF. Then HMFCA and DFF can be further oxidized into FFCA, while the generation of FDCA is inhibited. The incorporation of Au or Ru in PtRhPdIr to form quinary HEA nanoparticles facilitates the HMF oxidation to DFF and the further generation of FFCA and FDCA. If both Au and Ru are incorporated to form PtRhPdIrAuRu HEA, the HMF oxidation to HMFCA and FFCA oxidation to FDCA were promoted, leaving the forming of DFF and FFCA minimal.

The conventional HECs preparation methods require high-temperature treatment, which induce catalyst particles aggregation and result in forming micron-scale particles. The catalytic activity is restricted by limited exposed active sites and poor intrinsic activities. Wang et al. prepared oxygen vacancy-rich HEO nanosheets by low-temperature plasma method for HMFOR [44]. The technique endows the as-synthesized HEOs with high surface area and nanosheets morphologies with a size about 20 nm. Particularly, oxygen vacancies improve the intrinsic activity as demonstrated by comparisons of current density after normalized by ECSA and BET surface area. PtRuAgCoNi HEA nanoparticles with an average diameter of 9 nm was prepared by low-temperature solvothermal method (200 °C) [104]. During the preparation procedure, PVP was added to inhibit the nanoparticle overgrowth and aggregation. Afterward, PVP was replaced by oleylamine and the obtained nanoparticles were calcined at low temperature (180 °C) to get surfactants-free HEAs. The nanosized HEAs presented much higher HMF electrooxidation activity and FDCA selectivity than the commercial Pt/C catalyst. Morphology control is also crucial for reactants confinement and induce specific local environment. Liu et al. synthesized a series of 2D HEOs with single-crystalline and nanoplates morphology by calcinating the parent high-entropy basic carbonate salts (Fig. 6g). H_2_O and CO_2_ releases during high-temperature treatment create numerous penetrated mesopores with size of 3–10 nm [105]. Shen et al. synthesized hollow HE-LH array catalysts by MOF-templated method for HMF oxidation (Fig. 6h) [74]. Finite element simulations revealed hollow array morphology induces a strong local electric field over all of the shell, thus promotes protons accumulation across the shell. While for its solid counterpart, protons only accumulate around the nanotip of the solid array.

### HMF and FF Hydrogenation

The hydrogenation of carbonyl group in HMF produces BHMF, which is an important starting molecule for manufacturing biodegradable plastics, polyesters, drugs and polyurethane foam [122, 123]. Li et al. found the HMF conversion and BHMF selectivity on HEA catalyst outperformed those of monometallic catalysts and their mixture [50]. Both monometallic Fe and Cu exhibited low activity and selectivity. Though Co and Ni exhibited high BHMF selectivity (> 80%), the conversion of HMF remained considerably low. Ru can achieve complete conversion of HMF, while the yield of BHMF is negligible. The mixture of the five metals presented HMF conversion of 23.8% and BHMF selectivity of 56%, which are much lower than those of FeCoNiCuRu HEA. The silica supported FeCoNiCuRu HEA presented 98.5% conversion of HMF and 99% BHMF selectivity.

**Fig. 7:**
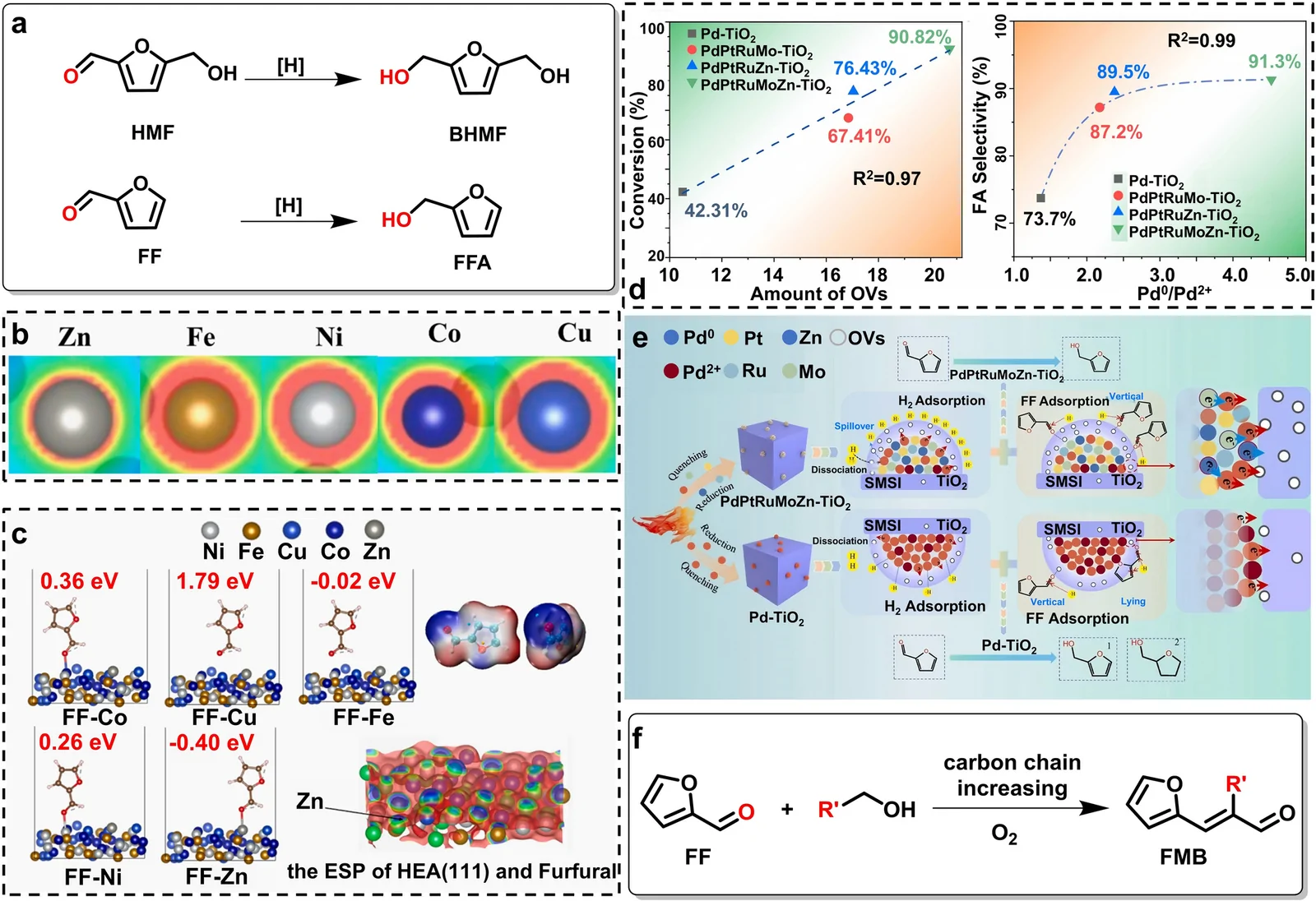
**a** FF and HMF hydrogenation to FFA and BHMF. **b** Charge density of metal in ultra-dilute NiCoCuZnFe HEA. Reproduced with permission [46]. Copyright 2023, Elsevier. **c** Adsorption of FF on the ultra-dilute NiCoCuZnFe HEA and the ESP of HEA and FF. Reproduced with permission [46]. Copyright 2023, Elsevier. **d** Correlation between amount of oxygen vacancies (Pd^0^/Pd^2+^) and FF conversion. Reproduced with permission [100]. Copyright 2024, Elsevier. **e** Reaction mechanism of FF hydrogenation to FFA in Pd-TiO_2_ and PdPtRuMoZn-TiO_2_. Reproduced with permission [100]. Copyright 2024, Elsevier. **f** Oxidative condensation of FF with alcohol

Similar to HMF, FF contains two functional groups, namely the furan ring and carbonyl group (C=O), which exhibit remarkable reactivity. The conversion of FF to furfuryl alcohol (FFA) is also achieved by C=O hydrogenation while preventing further hydrogenation of furan ring (Fig. 7a). The over hydrogenation will produce tetrahydrofuran alcohol (THFA). FFA emerges as a core fine chemical endowed with versatile applications spanning multiple industrial sectors, such as resin synthesis, coating manufacturing, and pharmaceutical production [124, 125].

Xu’s group synthesized NiCoCuZnFe HEA in H_2_ calcination atmosphere [46]. Zn sublimated from HEA core as calcination temperature elevated, thereby forming ultra-dilute HEAs (0.45% Zn). Due to electronegativity differences, electrons redistribute among the multi metals (Fig. 7b). The electron-rich Cu repelled the furan ring because of the strong repulsive force and made the adsorption configuration of FF turned into an oblique or vertical mode, thus inhibited the over hydrogenation. DFT calculation results showed Zn was the strongest adsorption sites of oxygen in aldehyde group (Fig. 7c). Other active metals dissociate hydrogen and transfer them to the aldehyde for selective hydrogenation. The ultra-dilute HEA exhibited 87.32% of FF conversion and 100% selectivity of FFA at 90 °C. The same group prepared ultra-low loading HEA PdPtRuMoZn supported on TiO_2_ catalyst (0.3 wt%) for highly active FF hydrogenation at ambient temperature [100]. Zn and Mo promoted the generation of oxygen vacancies through inducing lattice distortion and weakening the Ti–O bond. The abundant oxygen vacancies are beneficial for reactant adsorption. Moreover, multielectron transfers in HEA resulted in more Pd^0^, which provided more active site for hydrogen dissociation. Results found the FF conversion and FFA selectivity presented a positive relationship with oxygen vacancies and the Pd^0^/Pd^2+^ ratio, respectively (Fig. 7d). DRIFTS results proved FF only exists in vertical configuration in HEA-TiO_2_, and both vertical and lying configurations exist in Pd-TiO_2_, which led to the formation of THFA (Fig. 7e). They further prepared PtCoNiCuZn@NC via pyrolysis MOFs-template in H_2_/Ar atmosphere, which presented 94% FF conversion and 100% selectivity of FFA with isopropyl alcohol (IPA) as the hydrogen donor [79]. High-temperature calcination prompted Co and Ni to migrate out from HEA, thus forming a PtCuZn-rich and CoNi-rich phase.

Typically, FF and HMF hydrogenation operates under high-temperature and high-pressure on homogeneous catalyst with H_2_ as the hydrogen source. The process suffers from the drawbacks of harsh reaction condition, intricate separation processes and high energy consumption [126]. Electrocatalytic hydrogenation offers an alternative to FF and HMF hydrogenation, characterization by mild reaction condition and facile separation [127]. The hydrogen source comes from the dissociation of water, thereby eliminating the requirement of high-purity H_2_ supply. Pd-based HEA catalyst presented outstanding activity and stability in the electroreduction of FF to FFA [78]. The incorporation of Pd in FeCoNiCu significantly elevates the FF conversion rate and FFA selectivity.

### FF Oxidative Condensation

The properties of liquid fuels are intimately linked to their carbon atom content. Biomass-derived products obtained through deoxygenative routes generally feature a carbon number lower than that of gasoline and diesel [128]. With respect to furfural-based biofuels, the control of carbon chain length is exceedingly meaningful in improving their application quality. Benefiting from the distinctive furan ring and aldehyde functional group in its molecular architecture, FF can readily participate in reactions beyond hydrogenation reduction, such as condensation, selective oxidation, reductive amination, and oxidative esterification, which enables the synthesis of high value-added chemicals.

Tong’s group synthesized C_7_–C_17_ fuel precursor via the oxidative condensation of FF with C_2_–C_12_ aliphatic alcohols by HECs (Fig. 7f) [47, 109]. To screen the optimal catalyst compositions, 14 kinds of HEAs with 12 different elements combinations were synthesized for furfural oxidative condensation [47]. By comparing the activity and selectivity of HEA catalysts with different element combinations, it was found that the introduction of Mg, La, Mn, Fe, and Cu is crucial for achieving high catalytic performance. When n-butanol acted as the aliphatic alcohol, 99.7% of FF conversion and 93.8% selectivity of 2-(furan-2-yl-methylene) butanal (FMB) could be achieved on MgLaFeMnCu HEAs in the presence of O_2_. Afterward, a new HEO catalyst MnFeCoNiCuOx was synthesized by the same group, a more than 99.0% conversion of FF and 91.5% selectivity of FMB was obtained [109].

### Lignin Depolymerization

**Fig. 8:**
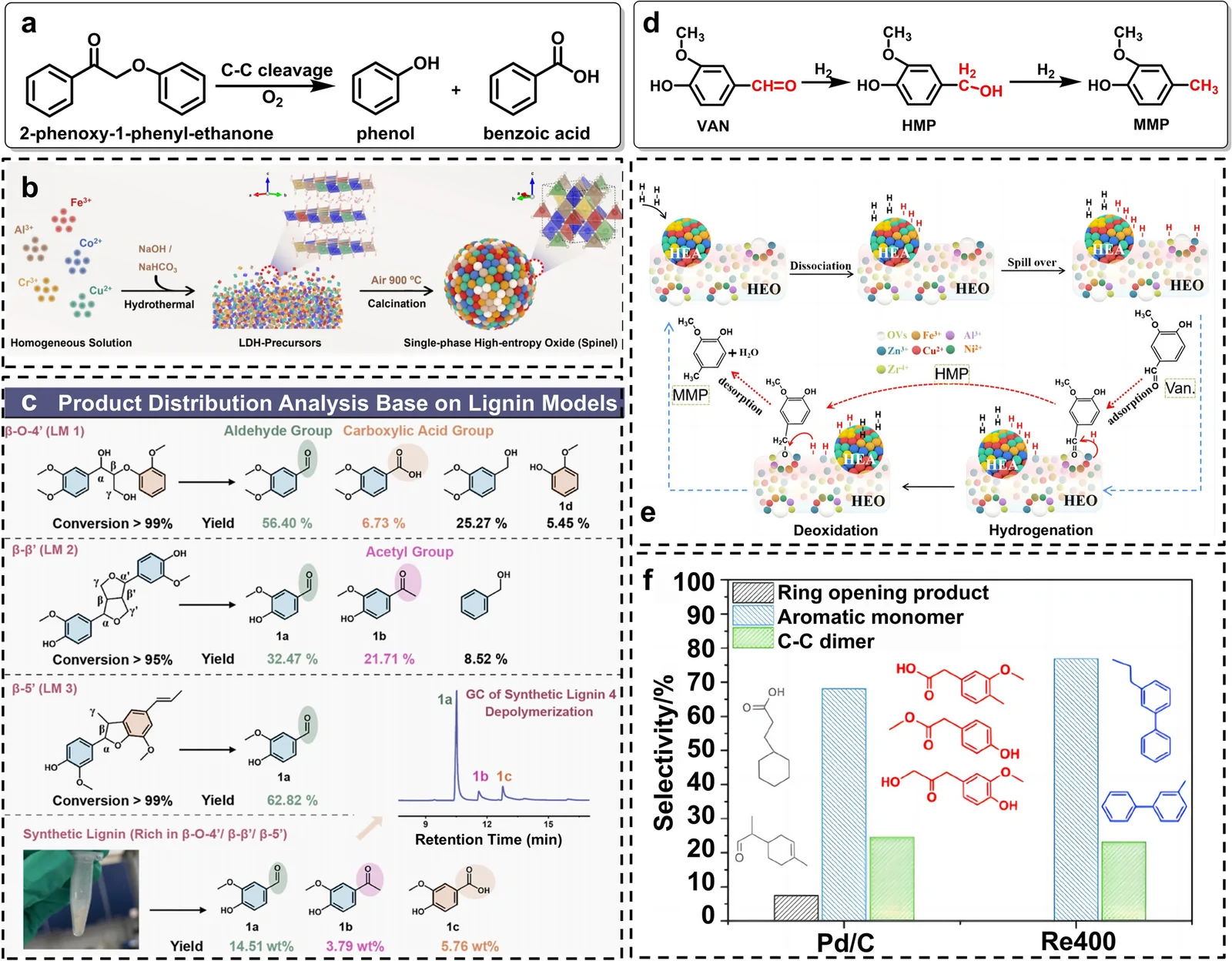
**a** Oxidative cleavage of 2-phenoxy-1-phenyl-ethanone. **b** Synthesis strategy of single-phase (AlCrFeCoCu)_3_O_4_ HEO catalysts. Reproduced with permission [52]. Copyright 2025, American Chemical Society. **c** Product distribution analysis based on lignin models and synthetic lignin. Reproduced with permission [52]. Copyright 2025, American Chemical Society. **d** Reaction pathway of VAN hydrodeoxygenation to MMP. **e** Selective hydrodeoxygenation reaction mechanism of VAN to MMP on HEA/HEO hetero-structured catalyst. Reproduced with permission [48]. Copyright 2023, John Wiley and Sons. **f** Product distribution of the lignin decomposition on Pd/C and Re400 (HEA/HEO hetero-structured catalyst). Reproduced with permission [48]. Copyright 2023, John Wiley and Sons

Lignin is the second most copious natural polymer, accounting for 30% of the organic carbon reservoir within the biosphere. Lignin is constituted by three distinct cinnamyl alcohol monomers, specifically p-coumaryl alcohol, coniferyl alcohol, and sinapyl alcohol, which linked by C–C and C–O bonds [129]. Lignin valorization requires the depolymerization process. There are two distinct methodologies (Fig. 8a): (1) oxidative depolymerization, which operates in the presence of oxygen to activate C–O and C–C bonds to yield aromatic aldehydes, ketones and acids, such as vanillin and syringaldehyde [130, 131]. (2) Reductive depolymerization, which requires a hydrogen source to cleave weaker C-O bonds, forming aromatic phenolic monomers [5].

Tong’s group synthesized HEO catalyst and calcinated it under H_2_ gas, the as-prepared MgCuMnFeCo-R catalyst possess numerous oxygen vacancies [88]. 2-Phenoxy-1-phenyl-ethanone was selected as a model chemical to explore the C–C bond oxidation cleavage performance. 94% of 2-phenoxy-1-phenyl-ethanone is converted into phenol (81.5% selectivity) and benzoic acid (94% selectivity) at 140 °C under 0.3 MPa of O_2_ for 4 h. Control experiments revealed alkaline earth metals plays a crucial role in C–C oxidation cleavage. Yu’s team synthesized (AlCrFeCoCu)_3_O_4_ HEO catalysts by calcinating LDH-precursors under high temperature (Fig. 8b) [52]. Lignin was fractionated by this catalyst into aromatic monomers with a 42 wt% yield, while the carboxylic acid production reached 150 wt% compared with the original hemicellulose content. Benefiting from the cocktail effect, the catalyst confers numerous oxygen vacancies, promoting oxygen adsorption and activation abilities for lignin oxidative depolymerization. Lignin models containing β-O-4, β-5, and β-β linkages were utilized to elucidate the mechanism (Fig. 8c). Results showed complete substrate conversion and C–O, C–C bonds cleavage during depolymerization.

Lignin oxidative depolymerization often generates products containing high oxygen contents. When used as biofuels, they suffer from the drawbacks of low energy density and unstable combustion. Selective hydrodeoxygenation reaction offers alternative to eliminate oxygen from lignin and preserves the benzene ring for getting aromatics products. For example, vanillin (VAN) is a typical lignin depolymerization derived product, its hydrodeoxygenation product 2-methoxy-4-methyl phenol (MMP) is an important chemical for synthesizing drugs [132]. The conversion of VAN to MMP involves two steps (Fig. 8d): (1) aldehyde hydrogenation generates hydroxyl groups, which converts VAN to 4-hydroxymethyl-2-methoxyphenol (HMP) and (2) hydroxyl deoxygenation generates alkyl, which converts HMP to MMP. Sun et al. constructed HEA/HEO hetero-structured catalyst for VAN hydrodeoxygenation, which exhibited 100% VAN conversion and 95% selectivity toward MMP [48]. HEA facilitates the hydrogen dissociation and the oxygen vacancies in HEO is beneficial for C=O adsorption (Fig. 8e). The hetero-structure interface offers a high-speed channel for hydrogen spillover which promotes the aldehyde hydrogenation and C-O dissociation to obtain the resulting MMP. Meanwhile, the HEA/HEO catalyst presented higher selectivity to aromatic monomer without forming ring-opening products in lignin reductive depolymerization reaction (Fig. 8f). The products distribution revealed the catalyst exhibited high activity in β-O-4 bond cleavage.

### GLU Conversion

**Fig. 9:**
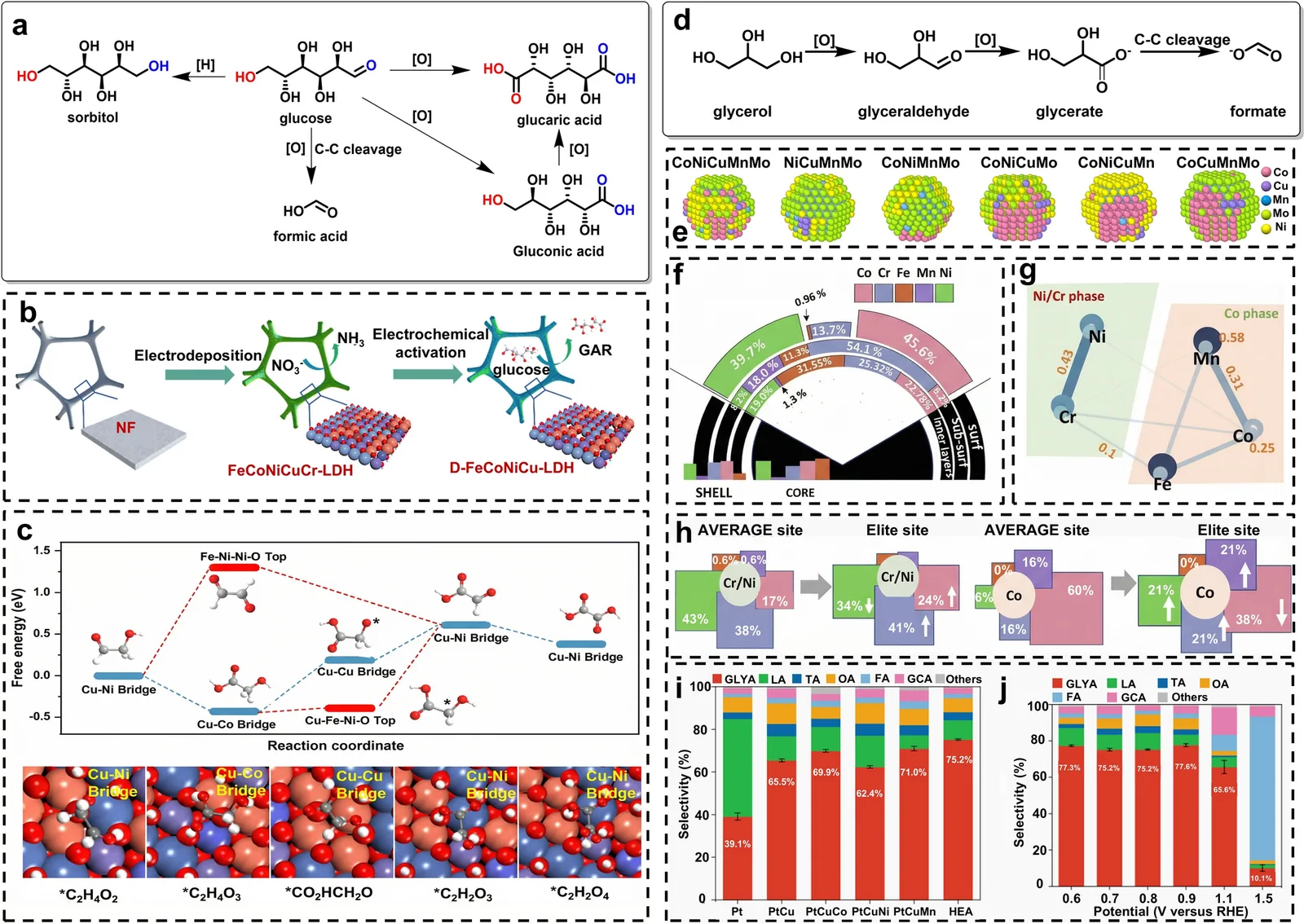
**a** Representative GLU valorization routes. **b** Synthesis procedure of defect-rich D-FeCoNiCu-LDH. Reproduced with permission [49]. Copyright 2024, Royal Society of Chemistry. **c** Reaction pathway for glycolaldehyde (*C_2_H_4_O_2_) to oxalic acid (*C_2_H_2_O_4_) and active adsorption sites for various reactants. Reproduced with permission [49]. Copyright 2024, Royal Society of Chemistry. **d** Reaction pathway of GLY oxidation reaction. **e** Optimized structures of quaternary and quinary alloys achieved by MC simulation. Reproduced with permission [45]. Copyright 2022, American Chemical Society. **f** Elemental spatial distribution in FeCoNiCrMnS_2_ HES nanoparticles. Reproduced with permission [65]. Copyright 2023, Elsevier. **g** Affinity between each elemental pair in FeCoNiCrMnS_2_. Reproduced with permission [65]. Copyright 2023, Elsevier. **h** Fraction for the coordination elements for Cr/Ni and Co phases. Reproduced with permission [65]. Copyright 2023, Elsevier. **i** Products distributions at 0.8 V vs. RHE in 1 M KOH with 0.1 M GLY over Pt, PtCu, PtCuCo, PtCuNi, PtCuMn, and PtCuCoNiMn HEA. Reproduced with permission [51]. Copyright 2025, Springer Nature. **j** Products distributions at various potentials in in 1 M KOH with 0.1 M GLY over PtCuCoNiMn HEA. Reproduced with permission [51]. Copyright 2025, Springer Nature

GLU is a simple C6 sugar with one aldehyde group and five hydroxyl groups. The GLU aldehyde hydrogenation produces sorbitol while its oxidation produces glyceric acid. If the terminal hydroxyl group is also oxidized to carboxyl group, glucaric acid is formed (Fig. 9a). The reactions necessitate aldehyde and (or) hydroxyl group oxidation while inhibiting C–C bond cleavage. Glucose can also be converted to formic acid with hydroxyl (aldehyde) oxidation and C–C cleavage.

Up to date, only GLU electrochemical oxidized to glucaric acid was reported on HECs. Glucaric acid is a top value-added compound that acts as a key intermediate for the production of biodegradable polymers, detergents, and metal complexation agents [133]. The multisites of HECs cooperatively catalyze the different reaction steps in a study by Wu et al. [49]. Defect-rich D-FeCoNiCu-LDH was fabricated using cyclic voltammetry to remove Cr on FeCoNiCuCr-LDH which exhibited 100% GLU conversion and more than 90% glucaric acid (Fig. 9b). Theoretical computations revealed that Cu-Co and Cu-Cu bridges promote dehydrogenation of the hydroxyl and hydrogen linked with carbon, respectively. Cu-Ni bridge facilitates the oxidation of aldehyde to carboxyl group (Fig. 9c).

### GLY Oxidation

GLY is a cheap by-product in biodiesel production and its further oxidation produces value-added chemicals such as glyceric acid, glycolic acid, and formic acid [134]. Among these products, formic acid has gained widespread recognition as a highly promising target compound, serving versatile roles as a chemical intermediate, hydrogen storage medium, and clean fuel [135].

GLY contains three active hydroxyl groups (-OH) in its molecular structure. In GLY oxidation process, hydroxyl is initially oxidized to aldehyde with the formation of glyceraldehyde. Then the consecutive oxidation of aldehyde yields glycolic acid. Glycolic acid undergoes C–C cleavage to form formic acid and glycolic acid. Glycolic acid is prone to decomposition and ultimately converts into two molecules of formic acid (Fig. 9d).

Wen’s team firstly investigated GLY oxidation to formate on HEA electrocatalyst [45]. In this pioneer work, machine learning combined with Monte Carlo simulation revealed surface elements tend to gather in CoNiCuMnMo-HEA and quaternary alloys, while the sublayer atoms do not (Fig. 9e). Mo and Ni atoms favor to locate on the surface layer, and Mn tends to locate at the sublayer. The mulit-element configurations make each site have different environment surroundings from their neighboring sites. Due to various intermediates could bind to different sites, it breaks the scaling relationship. The catalytic sites in CoNiCuMnMo-HEA are Mo sites coordinated by Mo, Mn, and Ni. Especially, this catalyst exhibited long-term stability over 12 days continuous electrolysis with 92% FE of formate during all round, demonstrating the high stability of HEAs. They further synthesized FeCoNiCrMnS_2_ HES nanoparticles and found the similar elemental spatial distribution nonuniformity phenomenon. Combined DFT calculation with Monte Carlo and machine learning simulations, it was found that Co and Ni tend to locate on the surface layer, Fe shows a strong tendency to distribute in the inner layer, while Cr, Mn, and Ni do not exhibit obvious distribution preferences (Fig. 9f). Metal atoms present different compatibilities, Ni tends to bind with Ni/Cr, while Co tends to bind with Co/Cr/Mn, thus forming two sperate phases: Ni/Cr and Co/Fe/Mn (Fig. 9g). In Cr/Ni sites, an increase in Co/Mn and a reduction in Ni is beneficial for producing high activity sites. However, a decrease in Co and an increase in Ni are favorable for Co sites (Fig. 9h). The catalyst particles achieve 90% FE of formate in the potential range of 1.2 to 1.45 V vs. RHE.

Guo et al. synthesized high-entropy selenides and observed severe lattice distortion [84]. The “lattice distortion effect” of the synthesized (CoNiCuMnMo)Se maintained an excellent GLY electrooxidation activity and 88% selectivity for formate. The internal lattice strain caused by different metal radii may provoke amorphous phase without forming phase separation. Xie and co-workers successfully fabricated a FeCrCoNiCu HE-LH, which possesses a distinctive hybrid structure combining quasi-single-crystalline and amorphous phases [111]. The ultrathin nanosheet HE-LH enriches abundant accessible high-valence metal sites, thereby presenting a superb formate FE of 92.9% in GLY electrooxidation reaction. Another study also found FeCoNiCuP HEA with nanosheet array morphologies presented remarkable glycerol electrooxidation activity and an impressive formate FE of 87.9% at 1.4 V vs. RHE, much higher than those of quaternary alloys [110].

As another value-added chemicals in GLY oxidation reaction, glycerate serves as a kind of food additive and precursors for manufacturing surfactants and biodegradable polymers [136]. The generation of glycerate from GLY faces significant challenge because the facile cleavage of C–C bonds. Pt exhibited only a 39.1% glycerate selectivity at 0.8 V vs. RHE. When Pt is alloyed with Cu, the glycerate selectivity surged to 65.5%. Furthermore, the incorporation of Co, Ni, and Mn into PtCu positively pushed the glycerate selectivity to 75.2% (Fig. 9i) [51]. In situ Raman and FTIR techniques were used to gain further insight into the reaction pathway. On the basis of in situ characterizations, the oxidation of GLY to glycerate involves the activation–dissociation of primary hydroxyl and the oxidation of hydroxyl to carboxyl via a glyceraldehyde intermediate. On pure Pt, glycerate and lactate are the predominant products. Alloying Pt with Cu that are more easily oxidized and interact strongly with the carbon atom hinders the migration of the hydroxyl group to the catalyst surface, thereby suppressing lactate generation. Further incorporation of other transition metals to form HEAs can fine-tune the electronic structure, thereby contributes to the achievement of ultimate performance. Characterizations revealed the HEA catalyst is electrochemically activated to form PtCu-rich core and PtCuCoNiMn surface structure. The incorporation of base metals induces electron accumulation at Pt sites, and they serve as buffer sites to protect the active Pt species from over oxidation. Compared with high formate selectivity system (Table 1), the optimized applied potential is much lower. Beyond 1.1 V vs. RHE, the high potential leads to the deep oxidation of GLY and formate is the predominant product at 1.5 V vs. RHE due to C–C cleavage (Fig. 9j).

## Challenges and Research Opportunities

This article comprehensively reviewed the most-updated research progress of HECs in various biomass-derived chemicals valorization reactions, covering the elemental composition, structure design strategies, and the reaction mechanisms. Through a critical review of the existing research, it is expected to provide a theoretical basis and breakthrough direction for the application of HECs in the field of biomass valorization in the future.

**Fig. 10:**
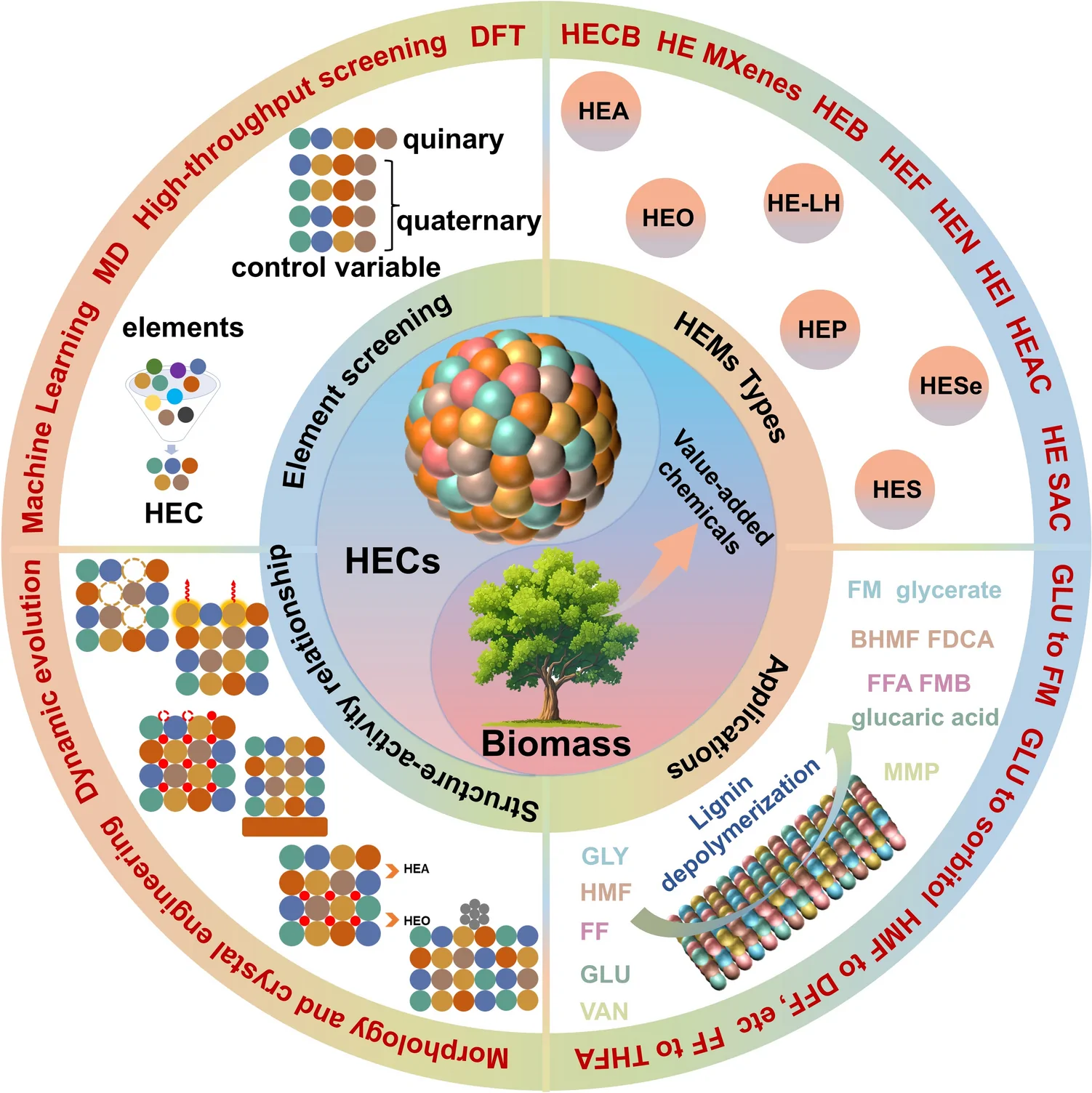
Research opportunities in designing highly efficient HECs for biomass-derived chemicals valorization

Despite the achievements, biomass-derived chemicals have diverse functional groups and their conversion covers a wide range of reactions and reaction types, which brings about both challenges and research opportunities in designing HECs. We outlined research opportunities in designing highly efficient HECs for biomass-derived chemicals valorization in Fig. 10.

### Precise Design and Synthesis of HECs with Targeted Properties

 The current design of HECs for biomass valorization lacks a systematic theoretical guidance system. The synergistic effect of multiple elements allowed HECs to exhibit outstanding catalytic performance in biomass conversion. However, when screening elements, the typical approach was to choose the frequently used elements from the previous published studies. The diversity of selectable elements results in a considerable number of element combinations. Taking HMF oxidation to FDCA as an example, there are about 20 metallic elements were chosen for constituting the HECs until now. The 20 elements enable the design of 15,504 quinary HEA combinations with 5 elements in equimolar ratios. The “trial–error method” relies on random or empirical parameter screening, which fails to efficiently narrow down the optimal constitution and preparation window, resulting in prolonged development cycles. High-throughput screening drastically cuts the trial-and-error costs, efficiently explores vast combinatorial spaces, accelerates optimal HEC discovery, and enables rational design instead of empirical guesswork [137, 138]. First, it fabricates diverse HEC libraries with varied elemental compositions/ratios via automated techniques. Then, high-throughput testing/characterization tools evaluate catalytic performance and microstructure. Finally, machine learning deciphers structure–activity relationships. The first step begins with fabricating a focused element library for specific reactions, which requires numerous literature support. For example, through an extensive literature analysis covering 14,242 publications, Chen et al. recognized 10 crucial active elements for HER, trimming the candidate pool to 126 Pt-based HEA combinations [139]. The publications about a specific biomass valorization reaction are still limited, far from satisfactory with building an element library.

The intricate compositional diversity and structural complexity inherent to HECs, coupled with the structural multifunctionality and chemical versatility of biomass-derived chemicals, pose considerable challenges to unraveling the underlying catalytic mechanisms in biomass valorization processes. Taking the electrooxidation of GLU as a typical case, GLU’s large molecular skeleton, multiple reactive sites, and complex conformational dynamics make theoretical calculations computationally prohibitive and inefficient. Thus, glycolaldehyde, a two-carbon molecule with one aldehyde and one hydroxyl group, sharing the same C/O ratio as GLU is adopted as a functional group surrogate to model the key dehydrogenation and oxidation steps of GLU’s aldehyde and hydroxyl groups [49]. While this strategy enables feasible theoretical exploration of core reaction mechanisms, it also introduces inherent limitations: (1) the surrogate molecules cannot reflect the steric hindrance effects of the parent biomass molecule’s large skeleton on active site adsorption and intermediate diffusion; (2) the absence of multiple vicinal reactive sites in surrogates fails to simulate the intramolecular electronic coupling and competitive reaction behaviors. These limitations lead to a gap between theoretical simulation results and actual catalytic performance of HECs for biomass valorization, hindering the accurate prediction of active site design and reaction pathway regulation. To address this obstacle, the integration of multiscale computational strategies with high-throughput screening and machine learning is a promising research direction.

### Expanding the HEC Types in Biomass-Derived Chemicals Valorization

 Initiated from the concept origin of HEA in 2004 and the high-entropy concept extended to HEO in 2015, HECs now cover a wide range of materials. Despite HEA, HEO, HES, HEP, HE-LH, HESe and hetero-structured HECs were applied in biomass valorization, some other emerging HEC categories are underexplored. These HEC types include HECBs, HENs, HEBs, HEFs, HEI catalysts, high-entropy Mxenes (HE Mxenes), high-entropy amorphous catalysts (HEACs), and HE SACs etc. These novel HECs possess unique electronic structures, tunable active sites, and strong resistance to harsh reaction conditions, which are ideal for the selective conversion of recalcitrant platform compounds. For example, the introduction of nitrogen into HENs engenders the formation of metal-nitrogen bonds, which modulates the electronic configuration of the catalyst via tailoring the electron density distribution and orbital hybridization degree of metal cations, thereby facilitating charge transfer dynamics and boosting electrical conductivity with favorable implications for catalytic efficacy [87]. The amorphous structure enables HEACs with no long-range order, which further eliminates the limitation of active sites caused by defects such as grain boundaries and dislocations, thus exposing more surface-active sites. Furthermore, HEACs exhibit a greater propensity for in situ reconstruction when exposed to electrochemical conditions, leading to the formation of more active species and a marked improvement in catalytic durability [140]. This characteristic endows HEACS with stronger adaptability to the adsorption and activation of different biomass-derived chemicals. HEIs feature regular atomic stacking and precisely positioned atomic sites, which construct unambiguous active site architectures and thus render them highly competent for intricate catalytic processes [141, 142].

### Expanding the Reaction Scope in Biomass Valorization

 More than 200 types of high-value chemicals can be synthesized from biomass and biomass-derived platform compounds. Up to now, HECs for biomass-derived chemicals valorization only covers HMF oxidation to FDCA, HMF and FF hydrogenation, FF oxidation condensation, lignin depolymerization, GLU to glucaric acid, and GLY oxidation to formate and glycerate. Expanding the application of HECs to more biomass valorization reactions and complex feedstocks is a key research direction. The multiple active sites synergy and abundant defects make HECs suitable for achieving high activity and selectivity toward target products. Taking HMF oxidation as an example, the FDCA selectivity is close to 100% owning to the consecutive aldehyde and hydroxyl groups oxidation to carboxylate is favorable. The HMF electrocatalytic oxidation usually follows route 1 because aldehyde is more active than hydroxyl, resulting DFF undetectable. How to design HEC catalysts to achieve highly selective oxidation of hydroxyl or one of the aldehyde groups, rather than ultimately being oxidized to carboxyl, is a challenging but significant issue. The achievement of this goal is conducive to the directed and highly selective generation of HMCA, DFF, and FFCA. HMF can react with alcohols to form HMF-derived ethers, which can be further transformed to alkyl levulinate [143, 144]. HMF can also be transformed into levulinic acid and formic acid by rehydration. FF can be converted to more than 80 value-added chemicals by reduction and oxidation [145]. For example, the hydrogenation of furan ring produces THFA, and the cleavage of C-O bonds produces furan and 2-methylfuran. GLU hydrogenation to sorbitol, and oxidation to glyceric acid and formic acid are also research hotspot [127, 146]. Apart from hydrogenation, hydrodeoxygenation, dehydrogenation and oxidation condensation, retro-aldol condensation is also an important reaction type in biomass valorization. Through retro-aldol condensation, GLU is able to produce erythrose and glycolaldehyde, and fructose can be converted to dihydroxyacetone and glyceraldehyde. The unexplored reactions and reactions types provide attracting application opportunities for HECs.

### Exploring More Structure Design Strategies and Their Relationship with Biomass Valorization Activity

 The complexity of HECs provides vast structure regulating space for optimizing specific reactions. As the application of HEMs in biomass valorization lags far behind other reactions, the structure design strategies that have been applied in other catalytic reactions can be transplanted to biomass valorization, and their correlations with catalytic performance deserve in-depth exploration. For example, HEA aerogels with highly distorted structure and 3D interconnected open channels presented efficient hydrogen oxidation reaction [147]. The retaining nanoscale properties at macroscale enable them presenting promising potential in biomass valorization. The size engineering of HECs is another strategy to optimize the available active sites for biomass valorization. Studies have found that there is a close correlation between particle size and catalytic performance within HECs [148]. Size modulation directly governs the exposure of active sites, lattice distortion degree, and mass transfer efficiency of HECs. Furthermore, size-induced high-entropy effect offers enhanced configurational diversity and entropy except from component-induced high-entropy effect [149]. HEAs typically forms FCC, BCC and HCP crystal structures, and HE ceramics (including HEOs, HENs, and HECs, etc.) typically follow rock-salt, perovskite, spinel, and fluorite crystalline structures. The atomic and electronic configurations can be tuned by crystal structure and exposed facets [150, 151]. Crystal engineering provides new design strategies for rational design of highly efficient HECs applied for biomass valorization and is a future focus. The structure dynamic evolution is a prevalent phenomenon in catalysis, which induces defect-rich surface and generates more active interfaces or species. In the electrochemical oxidation of small molecules such as HMF and GLY, the in situ reconstructed oxyhydroxides often act as the active sites. More in situ characterization methods should be used for providing mechanism insight.

## Abbreviations

HE: High-entropy
HEM: High-entropy material
HEC: High-entropy catalyst
HEA: High-entropy alloy
HEO: High-entropy oxide
HECB: High-entropy carbide
HEN: High-entropy nitride
HESe: High-entropy selenide
HES: High-entropy sulfide
HEP: High-entropy phosphide
HEB: High-entropy boride
HEF: High-entropy fluoride
IPA: Isopropyl alcohol
FE: Faradaic efficiency
GLU: Glucose
GRA: Glucaric acid
GLY: Glycerol
FM: Formate
OVs: Oxygen vacancies
HEACs: High-entropy amorphous catalysts
HE SACs: High-entropy single-atom catalysts
HE-LH: High-entropy layered hydroxides
FF: Furfural
FFA: Furfuryl alcohol
THFA: Tetrahydrofuran alcohol
FMB: 2-(Furan-2-yl-methylene) butanal
HMF: 5-Hydroxymethylfurfural
BHMF: 2,5-Furandimethanol
HMFCA: 5-Hydroxymethyl-2-furancarboxylic acid
DFF: 2,5-Diformylfuran
FFCA: Formyl-2-furancarboxylic acid
FDCA: 2,5-Furandicarboxylic acid
VAN: Vanillin
HMP: 4-Hydroxymethyl-2-methoxyphenol
MMP: 2-Methyl-4 methoxy phenol
DFT: Density functional theory
SMSI: Strong metal–support interaction
PDOS: Projected density of states
